# Lamins regulate cancer cell plasticity and chemosensitivity

**DOI:** 10.3389/fonc.2025.1599175

**Published:** 2025-07-10

**Authors:** Guofang Chen, Tingyi Wei, Ao Huang, Junwei Shen, Furong Ju, Shichao Huang, Haisen Li

**Affiliations:** ^1^ Shanghai Key Laboratory of Maternal Fetal Medicine, Clinical and Translational Research Center of Shanghai First Maternity and Infant Hospital, School of Medicine, Tongji University, Shanghai, China; ^2^ Ninth People’s Hospital, School of Medicine, Shanghai Jiao Tong University, Shanghai, China; ^3^ Shanghai Institute of Precision Medicine, Ninth People's Hospital, Shanghai Jiao Tong University, Shanghai, China; ^4^ Clinical Research Center for Mental Disorders, Shanghai Pudong New Area Mental Health Center, School of Medicine, Tongji University, Shanghai, China; ^5^ Department of Biomedical Sciences, Jockey Club College of Veterinary Medicine and Life Sciences, The Tung Biomedical Sciences Centre, City University of Hong Kong, Hong Kong, Hong Kong SAR, China; ^6^ State Key Laboratory of Cell Biology, Shanghai Institute of Biochemistry and Cell Biology, Center for Excellence in Molecular Cell Science, Chinese Academy of Sciences, Shanghai, China; ^7^ Zhongshan Hospital, Fudan University, Shanghai, China

**Keywords:** lamin, cancer cell plasticity, PD-L1, chemosensitivity, HIF-1 signaling

## Abstract

**Background:**

Stem cell plasticity plays key roles in mammalian organogenesis, tissue homeostasis, and carcinogenesis. Given its tolerance to anti-tumor therapy and its promotion on immunosuppressive microenvironment, cancer cell plasticity is a major contributor to cancer recurrence and metastasis. It is necessary to explore novel avenues to resolve the limitations of current treatments.

**Methods:**

We established stable cancer cell lines harboring all lamin knockdown and then explored the effects of all lamin deficiency on cancer plasticity and tumorigenesis in both cell and subcutaneous mouse models.

**Results:**

We found that all lamin knockdown disrupts cancer cell plasticity and impairs tumor progression. The deficiency of all lamin subtypes impaired the stemness and cell cycle transition of cancer cell. Lamin knockdown modulated genomic damage and repair pathways, inhibited mitochondrial function, and triggered cellular senescence. Moreover, lamin knockdown within cancer cell suppressed cancer growth *in vivo* by enhancing the infiltration and activation of functional T cells. Mechanistically, lamin knockdown reduced the expression of inhibitory immune checkpoints and inflammatory factors in cancer cell via the HIF-1 signaling pathway, which led to the increased sensitivity of cancer cells to chemotherapy.

**Conclusions:**

Overall, our findings characterize the significance of nuclear lamins in cancer cell plasticity and offer an attractive way to improve the effectiveness of anti-cancer therapy.

## Introduction

The lamin organizes into a filamentous matrix beneath the nuclear envelope and is a central hub for the nuclear lamina in eukaryotic cells (1, 2). The nuclear lamina serves as the meshwork to support nuclear envelope, maintain nuclear shape, benefit chromatin organization, and regulate gene expression. Thus, the lamin is essential for nuclear integrity and three-dimensional chromatin organization and functions as a crucial regulator in nuclear architecture, chromatin orientation, gene transcription, signal transduction, and epigenetic modification (3–6). Mammalian lamins comprise two evolutionarily conserved subtypes: A-type lamin (LMNA) and B-type lamin (LMNB1 and LMNB2). The mutations or alterations in these proteins have been associated with diverse pathologies including premature aging syndromes, cardiomyopathy, metabolic disorders, and multiple malignancies (7–16). Specifically, LMNA knockdown has been implicated in suppressing the proliferation and migration of human renal carcinoma and prostate cancer cells through the regulation of PI3K/AKT pathway (17, 18). LMNB1 ablation has been described to inhibit the viability and invasion of human prostate cancer cells by reducing FOXD1 expression (19), whereas LMNB2 deletion impedes the *in vitro* and *in vivo* growth of human colorectal cancer cell or bladder cancer cell via upregulating P21 expression or inhibiting CDCA3 transcription respectively (7, 20). Likewise, depleting B-type lamin has been shown to impair the growth of human pancreatic adenocarcinoma and esophageal cancer cell both *in vitro* and in subcutaneous mouse models (15, 21), among which the molecular mechanisms are yet to be elucidated. It is feasible in theory that specific lamin loss may be a therapeutic strategy for a given cancer. Notably, the relationship between lamin proteins and cancer is complex and could be influenced by many factors such as cancer type, stage, and genetic background. Up to now, it is unclear how the knockdown of all lamin subtypes modulates cancer occurrence and progression. Growing evidence indicates that a small subpopulation of cancer cells known as cancer stem-like cell is responsible for tumor initiation, progression, and recurrence due to their self-renewal and plasticity (22–25). Therapeutic resistance driven by this cell population remains a major barrier for curative outcomes, so it is necessary to develop novel ways for the elimination of resistant cancer stem-like cells during cancer therapy. However, whether the lamin network is crucial in cancer cell stemness and plasticity remains poorly understood.

In the present study, we determined the roles of nuclear lamins in cancer stemness and tumorigenesis. The lamin proteins were essential for cancer cell plasticity, and their knockdown resulted in the impairment of tumor growth and the enhancement of chemosensitivity.

## Materials and methods

### Plasmid construction and lentivirus generation

The shRNAs were synthesized as DNA oligos and then subcloned into the pLKO.1 expression plasmid (8453, Addgene). The lentivirus particles were generated by the co-transfection of pLKO.1 and two helper (pVSVG and pMD2.G) plasmids into human embryonic kidney 293T (HEK293T) cells (CRL-11268, ATCC) using the polyethylenimine reagent (101000029, Polyplus). The sequence of shRNAs is provided in **Supplementary Table 1**.

### Cell culture and transfection

The HEK293T cells were cultured in DMEM (11965-065, Thermo Fisher Scientific) containing 10% fetal bovine serum (FBS) (10099141C, Thermo Fisher Scientific) and 1% penicillin/streptomycin (15140122, Thermo Fisher Scientific). The colorectal carcinoma CT26 cells (CRL-2638, ATCC) were maintained in RPMI-1640 medium (11875093, Thermo Fisher Scientific) containing 10% FBS and 1% penicillin/streptomycin. For stable cell generation, lentivirus particles containing the control or lamin shRNA were incubated with the cells for 72 hours, which were then selected by 5 μg/mL puromycin (A1113803, Thermo Fisher Scientific) for one week. The desired cells were expanded later and cultured in 5 μg/mL puromycin. Murine macrophage cell line RAW264.7 (TIB-71, ATCC) and mammary cancer cell 4T1 (CRL-2539, ATCC) were also cultured in RPMI-1640 medium containing 10% FBS and 1% penicillin/streptomycin. All cells were cultured at 37°C with 5% CO2. To explore the downstream pathway underlying lamin knockdown, control and lamin-deficient cells were seeded into 12-well culture plates at a density of 1.5 × 10^5^ per well, and then treated with the small-molecule inhibitor of selective pathways. These inhibitors were listed as below: BAY872243 (SC1193, Beyotime), FR180204 (HY-12275, MedChemExpress), GSK8612 (HY-111941, MedChemExpress), Sorafenib (HY-10201, MedChemExpress), and/or Rosiglitazone (HY-17386, MedChemExpress).

### *In vivo* tumor models

The animal research conducted in this study was in accordance with the Scientific and Ethical Committee of the Shanghai First Maternity and Infant Hospital, School of Medicine, Tongji University. Four-week-old BALB/c and BALB/cNj-Foxn1nu/Gpt mice were purchased from GemPharmatech (Nanjing, China) and then housed in a constant temperature (24-26°C) and humidity (40-60%) room with a 12-hour light-dark cycle. The cancer cells without or with lamin knockdown were subcutaneously injected into the left side of 5-week-old mice at a dosage of 5 × 10^5^ cells/mice. For measuring the responsiveness of lamin-deficient tumor to clinically anticancer drugs, 10 mg/kg sorafenib was infused daily into the mice via intragastric administration from posttransplant day 8. The tumor volumes were periodically determined with a manual caliper and calculated using the below formula: ½ × longitudinal diameter (length) × the greatest transverse diameter (width)^2^. When their volume reached 2000 mm^3^, the tumors were harvested for further analysis.

### Colony formation assay

The cells without or with lamin were plated in a 6-well culture plate at a density of 1 × 10^3^/well and then cultured at 37°C, 5% CO_2_ for 2 weeks. These cells were stained with crystal violet and then visualized using an Olympus CK30-F200 microscope.

### Single-cell tracking assay

PerkinElmer 96-well culture plate was coated with the laminin at a concentration of 1μg/cm^2^ for 24 hours. Single cell population was seeded into the above 96-well plate at a density of 1 × 10^4^/well and then maintained at 37°C for 120 hours. Cell behaviors were scanned and imaged every 4 hours using the Livecyte^®^ Kinetic Cytomete (phasefocus) equipped with a sCMOS camera and 20×Plan Phase objective (Olympus). The cell index was calculated with Livecyte software after normalization to the initial image.

### EdU proliferation assay

This EdU proliferation assay was measured using BeyoClick™ EdU-555 kit (C0075S, Beyotime). EdU is a thymidine analog and labels the dividing cell by incorporating into nascent DNA during the S-phase. The EdU solution was added into the culture medium at a final concentration of 10 μM following the manufacturer’s instruction. Two hours later, the cells were washed with PBS (C0221A, Beyotime) and then fixed in 4% paraformaldehyde (P0099, Beyotime) for 15 minutes (mins). After their permeabilization with 0.3% Triton X-100, the cells were incubated in Click Additive Solution for 30 mins at room temperature (RT). In the end, DAPI was added and treated for 10 mins. The cells were imaged using an Olympus BX63F microscope.

### Cell cycle and apoptotic analyses

Cancer cells were digested into single cell and then resuspended in fresh culture medium. Hoechst33342 (C1022, Beyotime) was added into the medium at a final concentration of 10 μg/ml and then incubated at 37°C for 45 mins. After three washes, the cells were run on the BD Fortessa machine. Apoptosis assay was performed following the manufacturer’s protocol (#C1062S, Boomtime). The data were analyzed using the FlowJo.

### FACS analyses of cancer cell and tumor

For surface marker analysis, cancer or RAW264.7 cells were suspended in cell staining buffer (FXP005, 4abio) and stained with specific fluorochrome-conjugated antibodies at appropriate concentrations as previous reports (26, 27). For the engrafted tumor, a portion of tumors were digested into single cells, filtrated through 70 μm cell strainers, and then resuspended in cell staining buffer. Zombie NIR Fixable kit (423105, Biolegend) was used to determine live or dead cells. Tumor cells were incubated with fluorochrome-conjugated antibodies at RT for 1 hour. These antibodies were listed below: APC anti-mouse CD3 (100236, Biolegend); FITC anti-mouse CD4 (100405, Biolegend); Brilliant Violet 421 anti-mouse CD8 (100737, Biolegend); PE anti-mouse CD69 (104507, Biolegend); APC anti-mouse PD1 (135210, Biolegend); FITC anti-mouse CD44 (156008, Biolegend); PE anti-mouse CD133 (141203, Biolegend); Brilliant Violet 421 anti-mouse CD47 (127527, Biolegend); PE anti-mouse H-2Kb (116508, Biolegend); APC anti-mouse PD-L1 (124311, Biolegend); FITC anti-mouse CD80 (104705, Biolegend); PE/Cy7 anti-mouse CD86 (105014, Biolegend). Following three washes, the stained cells were analyzed on BD Fortessa machine (BD Biosciences), and the data were processed with the FlowJo software.

### Immunohistochemical analysis

The tumors were washed with PBS and then fixed in 4% paraformaldehyde overnight at 4°C. The samples were embedded in the paraffin tissue blocks, cut into 10-μm sections, and then transferred onto Bond-Rite™ slides. After being heated at 60°C for 60 mins, the sections were deparaffinized in fresh xylene and then rehydrated with descending grades of alcohol. The sections were soaked in blocking buffer (P0260, Beyotime) for 20 mins and then stained with primary antibodies at 4°C overnight. These primary antibodies were listed below: Rabbit anti-mouse CD3 (78588, Cell Signaling Technology); Rabbit anti-mouse CD8 (98941, Cell Signaling Technology); Rabbit anti-mouse STING (13647, Cell Signaling Technology); Rabbit anti-mouse Ki67 (12202, Cell Signaling Technology). After three washes, the sections were successively incubated with secondary antibody and DAPI for 3 hours. The coverslips were then applied with permanent synthetic mounting media. All pictures were viewed and taken under an inverted microscope.

### Western blotting

Cells were dissociated in RIPA lysis buffer (P0013B, Beyotime) containing proteinase inhibitor cocktail. After protein concentration was determined, total proteins were separated on SDS-PAGE gels, separated at 200V for 1 hour, and transferred onto the PVDF membranes. The membranes were blocked in 5% non-fat milk/TBST buffer and then incubated overnight with primary antibodies as previous reports (28, 29). Primary antibodies were listed below: NANOG (A22625, ABclonal), ALDH1A1 (A0157, ABclonal), DDB1 (A2896, ABclonal), Ku70 (A0883, ABclonal), Ku80 (A5862, ABclonal), RAD50 (A3078, ABclonal), RAD51 (A2829, ABclonal), BRCA1 (A11034, ABclonal), NBS1 (7703, ABclonal), DDB1 (A2896, ABclonal), DDB2 (A1848, ABclonal), Tubulin (11224-1-AP, Proteintech), INO80 (ab105451, Abcam), MRE11 (A2559, ABclonal), DDB2 (A1848, ABclonal), Rad23B (A1034, ABclonal), XPC (A8354, ABclonal), Phospho-STING (50907, Cell Signaling Technology), SDHA (11998, Cell Signaling Technology), PHB1 (2426, Cell Signaling Technology), VDAC (4661, Cell Signaling Technology), HSP60 (12165, Cell Signaling Technology), COX IV (4850, Cell Signaling Technology), Vinculin (4650, Cell Signaling Technology), ERK1/2 (4695, Cell Signaling Technology), Phospho-ERK1/2 (4370, Cell Signaling Technology), IL-1β (A16288, ABclonal), NLRP3 (15101, Cell Signaling Technology), TBK1 (38066, Cell Signaling Technology), Phospho-TBK1 (5483, Cell Signaling Technology), cGAS (15102, Cell Signaling Technology), Phospho-STING (50907, Cell Signaling Technology), STING (19851-1-AP, Proteintech), Phospho-STAT1 (8826, Cell Signaling Technology), Phospho-STAT3 (9145, Cell Signaling Technology), PPAR-γ (2443, Cell Signaling Technology), Phospho-P62 (13121, Cell Signaling Technology), LMNA (AF7350, Beyotime), LMNB1 (AF1408, Beyotime), LMNB2 (AF0219, Beyotime). Following three washes, the membranes were probed with HRP-conjugated secondary antibodies at RT for 2 hours. The target proteins were viewed with an ECL Plus Kit (Abcam). ImageJ software was used to view protein intensity.

### RNA isolation and RT-PCR

Total mRNAs were purified from the cells with Trizol reagent kit (Invitrogen, USA) according to the manufacturer’s instructions. They were reversely transcribed into cDNAs using the HiScript II Q Select RT SuperMix kit (R232-01, Vazyme). The reaction of quantitative PCR was performed with SYBR Green PCR Master Mix (Q111-02, Vazyme). The 2^-ΔΔCt^ method was used to calculate the relative expression level of target genes after normalization to the internal gene *GAPDH.* The sequences of RT-PCR primers are listed in the **Supplementary Table 2**.

### RNA sequencing

Total RNA quality was determined using Agilent 2100 Bioanalyzer (Agilent Technologies, USA). Eukaryotic mRNA was enriched by Oligo(dT) beads, fragmented into short fragments, and reversely transcribed into cDNA using the Next Ultra RNA Library Prep Kit for Illumina (7530, NEB). These double-stranded cDNA fragments were added with A-base, ligated to sequencing adapters, and then purified with the AMPure XP Beads. The cDNA library was sequenced using Illumina Novaseq 6000 by Gene Denovo Biotechnology Co. (Guangzhou, China).

### Immunofluorescence staining

Cancer cells without or with lamin knockdown were washed two times in PBS and then changed with fresh culture medium containing 10 μg/ml Hoechst33342. One hour later, the cells were washed with culture medium and viewed under the Leica TCS/SP8 microscope.

### Statistical analysis

Quantification was derived from at least three independent experiments and shown as mean ± SEM. The statistical significance was determined by Student’s t-test or by one-way analysis of variance (ANOVA). P-value of less than 0.05 was thought to be significant.

## Results

### Lamins are highly expressed in many cancers and essential for cancer cell stemness

**Figure 1:**
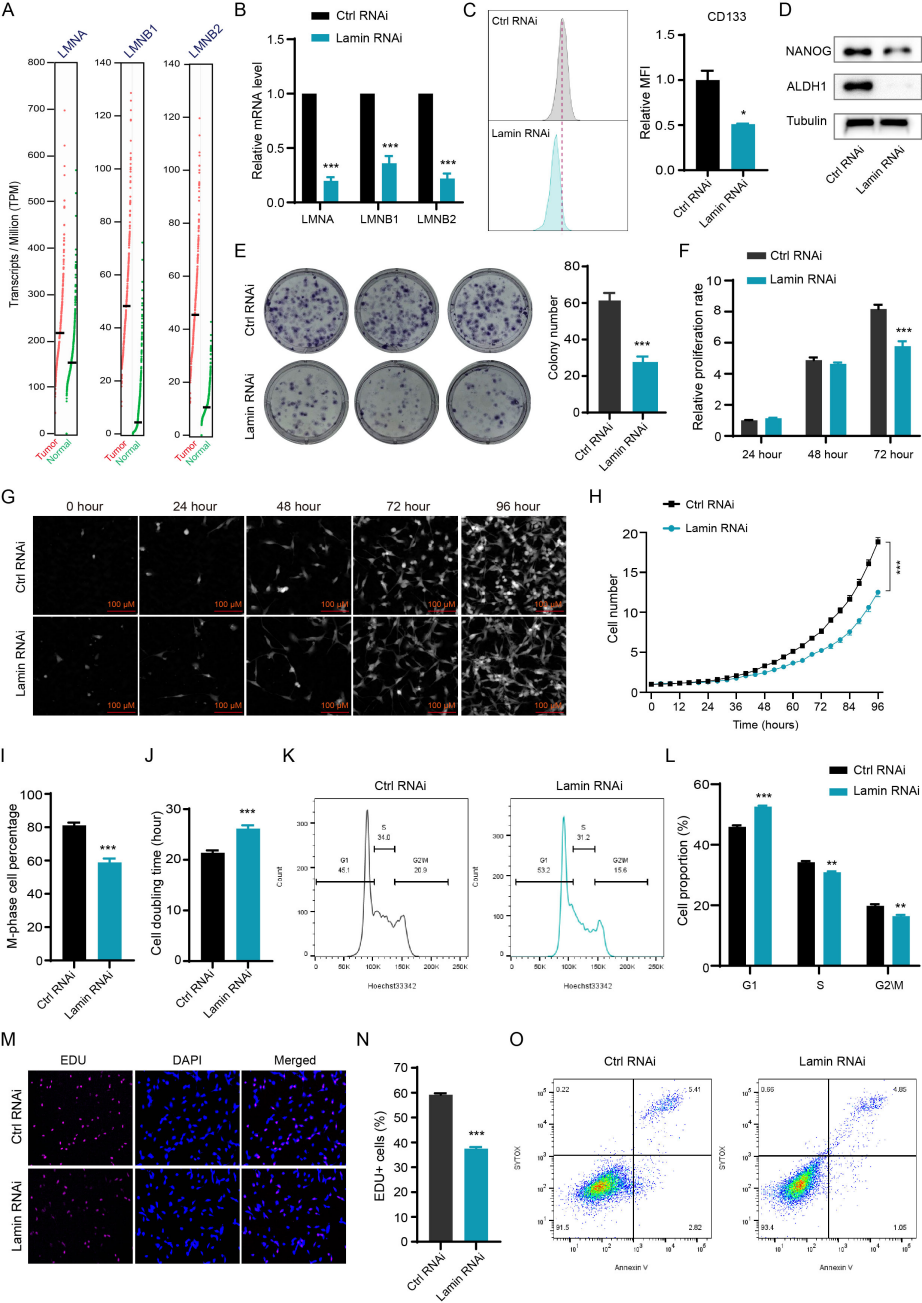
Lamin deficiency reduces cancer cell plasticity and proliferation. **(A)** Comparison of the levels of LMNA, LMNB1, and LMNB2 transcripts in the COAD and normal tissues from the TCGA database. **(B)** Q-PCR analysis of *LMNA*, *LMNB1*, and *LMNB2* mRNA levels in cancer cells without or with lamin deficiency. **(C)** FACS determination of CD133 expression level in the control and lamin-deficient cancer stem-like cell. Right: Quantification of relative CD133 fluorescence intensity; Left: Representative FACS histograms. **(D)** Western blotting analysis of pluripotent marker NANOG and ALDH1 levels in the control and lamin-deficient cell. **(E)** Colony formation assay. Left: Representative colony images; Right: Quantification of colony numbers. **(F)** CCK8 assay for relative proliferation rate of control and lamin-deficient cell. **(G)** Representative images of single-cell tracking assay. Single cancer stem-like cell without or with lamin deficiency was traced continuously for 96 hours. **(H)** Quantification of single cell proliferation rate. **(I)** Quantification of the percentage of M-phase cells at the 96-hour time point. **(J)** Quantification of the doubling time of the control or lamin-deficient cell. **(K)** Representative FACS plots of cell cycle distributions in the control or lamin-deficient cancer cell. **(L)** Quantification of the percentages of different cell cycle phases in the control and lamin-deficient cancer cells. **(M)** Representative staining pictures of the EdU assay. **(N)** Quantification of EdU^+^ cell percentage. **(O)** Representative FACS plots of apoptotic assay in cancer cells without or with lamin deficiency. Quantification is shown as mean ± SEM (n = 3). *P < 0.05, **P < 0.01, ***P< 0.001 versus the control RNAi.

To evaluate the pathological significance of lamin in tumor progression, we analyzed the expression profiles of lamin in various human cancers via the cBioPortal Cancer Genomics database. The expression levels of *LMNA*, *LMNB1*, and *LMNB2* mRNAs were higher in most tumors than in normal adjacent tissues, such as bladder urothelial carcinoma (BLCA), colon adenocarcinoma (COAD), kidney renal clear cell carcinoma (KIRC), liver hepatocellular carcinoma (LIHC), pancreatic adenocarcinoma (PAAD), and thyroid carcinoma (THCA) (**Supplementary Figures 1A-C**). The transcriptome analysis showed that the COAD tumors had more lamin transcripts than normal tissue (**Figure 1A**), supporting the upregulation of LMNA, LMNB1, and LMNB2 expressions in tumor tissues. The COAD patients with low lamin expression likely presented better survival outcomes in comparison to the patients bearing high lamin level (**Supplementary Figure 1D**). To explore if lamin is essential in cancer cell plasticity, the CD44^+^ CD133^+^ cell population was sorted out from murine CT26 cancer cell and then applied in the below experiments. Both CD44 and CD133 are widely used surface markers for cancer stem-like cells (22, 30, 31). These cancer cells with stem-cell-like properties were engineered for the simultaneous knockdown of three lamin subtypes via the shRNA-mediated knockdown system. The knockdown efficiencies of LMNA, LMNB1, and LMNB2 were confirmed at both transcript and protein levels (**Figure 1B**, **Supplementary Figure 2A**). The expression of stemness marker CD133 was significantly decreased by lamin knockdown (**Figure 1C**). Consistently, lamin knockdown reduced the levels of pluripotent markers ALDH1 and NANOG (**Figure 1D**), indicating the stemness reduction after lamin knockdown. In line with this notion, lamin knockdown suppressed the colony formation ability of single cancer cell (**Figure 1E**). The CCK8 assay showed that lamin-deficient cell population exhibited a lower proliferation rate in comparison to the control cells (**Figure 1F**). Single-cell tracking assay revealed that lamin-deficient cell had a much weaker proliferative capacity than the control cell (**Figures 1G, H**), leading to the fewer cell number and the lower M-phase cell percentage at 96-hour time point (**Figures 1H, I**). Moreover, a single lamin-deficient cell needed more time to finish its double period, as demonstrated by the higher doubling time of lamin-deficient cell than the control cell (**Figure 1J**). Additionally, cell cycle analysis showed that lamin knockdown gave rise to the increase of the G1 phase and the reduction of S and G2/M phases (**Figures 1K, L**). Lamin knockdown resulted in the notable reduction of EdU^+^ cells (**Figures 1M, N**), suggesting the inhibition of lamin deficiency on cancer cell proliferation. However, lamin knockdown was found to decrease cell apoptosis, as evidenced by the lower percentages of Annexin-V^+^ or CASP3^+^ cells (**Figure 1O**, **Supplementary Figure 2B**). Thus, the inhibition of lamin knockdown on cancer cell proliferation and stemness was not due to the reduced apoptosis. Lamin-deficient cancer cell was shown to display anomalous nuclear shape in comparison to the round nuclear phenotype of control cell (**Supplementary Figure 2C**), indicating that all lamin knockdown might trigger nuclear anomaly. Collectively, these data suggest that lamin had higher expression levels in many cancers and its knockdown inhibited cancer cell stemness and proliferation.

### Lamin-deficient cancer cells presented abnormal DNA repair capacity and mitochondrial function

**Figure 2:**
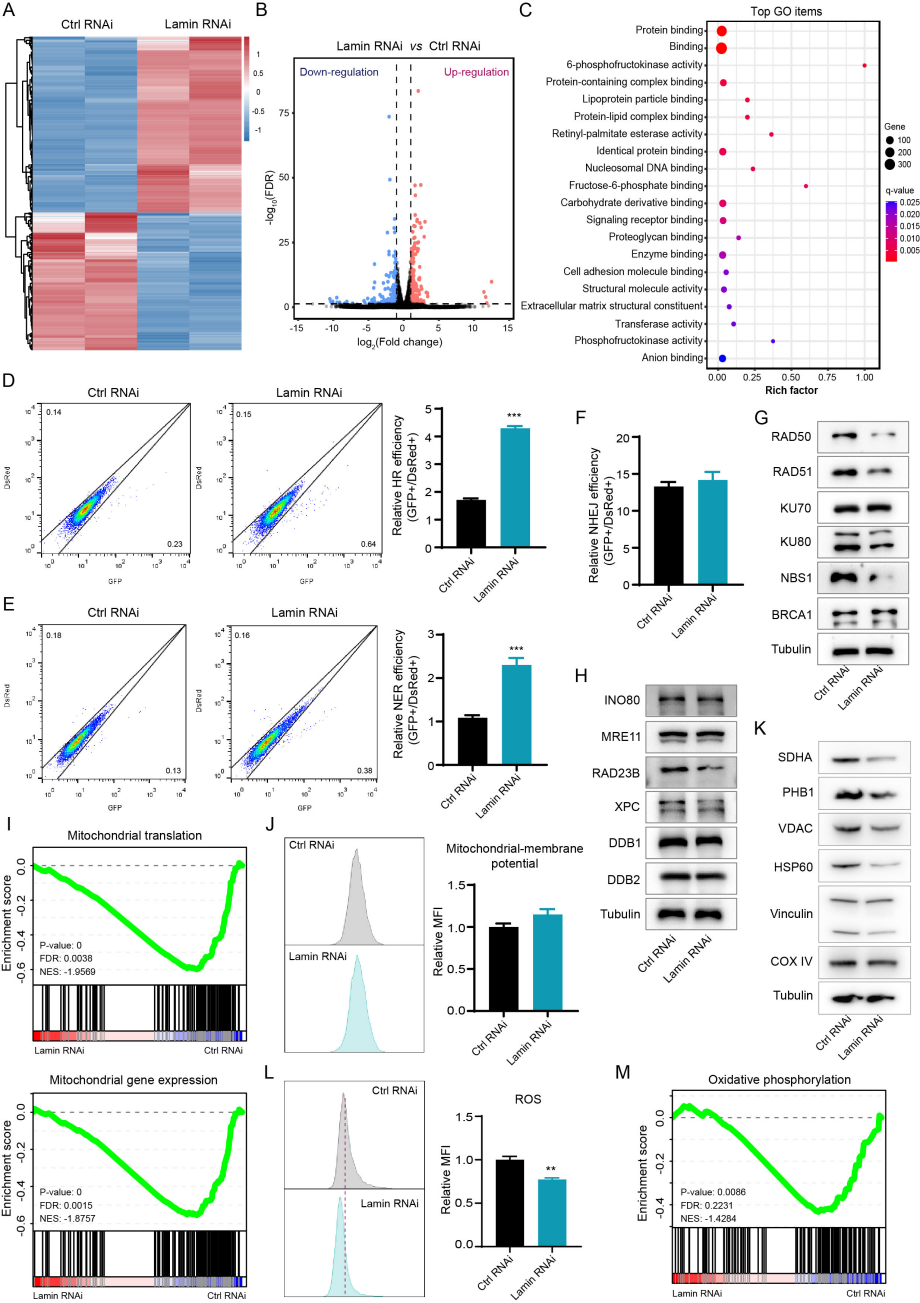
Lamin deficiency modulates genomic integrity and mitochondrial function. **(A)** Heatmap of gene expression profiles in cancer stem-like cells without or with lamin deficiency. **(B)** The classification of differentially expressed genes. Downregulated and upregulated genes are shown in blue and red, respectively. **(C)** Gene Ontology (GO) analysis of differentially expressed genes in the RNA-seq data. **(D)** FACS assessment of HR efficiency in the control and lamin-deficient cell. Left: Representative FACS plots; Right: Quantification of relative HR efficiency. **(E)** FACS assessment of NER efficiency in the control and lamin-deficient cell. Left: Representative FACS plots; Right: Quantification of relative NER efficiency. **(F)** Assessment of NHEJ efficiency in the control and lamin-deficient cell. **(G)** Western blotting analysis of repair-related proteins in cancer cells without or with lamin deficiency. **(H)** Western blotting analysis of additional repair-related proteins in the control and lamin-deficient cell. **(I)** GSEA analyses of mitochondrial translation and mitochondrial gene expression signatures. FDR: False discovery rate; NES: Normalized enrichment score. **(J)** FACS analysis of mitochondrial membrane potential in cancer cell without or with lamin knockdown. Left: Representative FACS plots; Right: Quantification of relative fold change of mitochondria. **(K)** Western blotting analysis of mitochondria-related proteins in the control and lamin-deficient cancer cell. **(L)** FACS analysis of intracellular ROS level in the control and lamin-deficient cancer cell. Left: Representative FACS histograms; Right: Relative fold change of ROS intensity. **(M)** GSEA analysis of the oxidative phosphorylation in the above RNA-seq data. Quantification is shown as mean ± SEM (n = 3). **P < 0.01, ***P< 0.001 versus the control RNAi.

To investigate how lamin deficiency affects cellular processes, we conducted transcriptome sequencing without or with lamin knockdown. Bioinformatic analysis showed that lamin knockdown changed the expression profiles of many functional genes, resulting in the downregulation or upregulation of 2667 or 1570 differently expressed genes (fold change ≥ 2) respectively (**Figures 2A, B**). A substantial number of the differently expressed genes were enriched in the processes, including but not limited to DNA binding, protein binding, transferase activity, phosphofructokinase activity, and biosynthetic and metabolic regulation (**Figure 2C**, **Supplementary Figure 2D**). Since DNA-binding proteins have critical roles in DNA repair pathways, we next tried to measure the influence of lamin knockdown on the genomic repair process. A sensitive fluorescent reporter assay, in which the I-SceI endonuclease creates genomic damage at the target locus (32, 33), was employed to determine the efficiency of homologous recombination (HR), non-homologous end joining (NHEJ), or nucleotide excision repair (NER). The successful repairment of genomic damage by the above DNA repair pathway led to the expression of the relative GFP reporter. Lamin knockdown was found to increase the repair efficiency of HR and NER pathways, as evidenced by the higher GFP^+^ cell proportion in lamin-deficient cells (**Figures 2D, E**). However, lamin deficiency did not affect the efficiency of the NHEJ repair pathway (**Figure 2F**). Western blotting analysis revealed that lamin knockdown led to the selective decrease in the levels of repair-related factors RAD50, RAD51, Ku80, NBS1, and RAD23B (**Figure 2G**). Conversely, other repair-associated proteins such as INO80 and MRE11 remained unchanged (**Figure 2H**). It is notable that lamin-deficient cancer cells might possess abnormal cellular components such as intracellular organelles (**Supplementary Figure 2E**). Given the importance of mitochondria in cellular metabolism and energy production, we then assessed the impact of lamin loss on mitochondrial function. GSEA analysis revealed that both mitochondrial gene expression and protein translation were suppressed by lamin knockdown (**Figure 2I**). Although having no affection on mitochondrial membrane potential (**Figure 2J**), lamin knockdown reduced the expression levels of functionally mitochondrial proteins SDHA, PHB1, VDAC, and HSP60 (**Figure 2K**). This observation suggests that mitochondrial activity in lamin-deficient cells was lower than in the control cells. Lamin-deficient cells were found to display less reactive oxygen species (ROS) than the control cell, as demonstrated by the lower fluorescent staining intensity in FACS assays (**Figures 2L**). Among the changed metabolic processes after lamin knockdown (**Supplementary Figure 2D**), the oxidative phosphorylation was likely decreased by lamin knockdown (**Figure 2M**). Nevertheless, the amount of oxidized lipids in the lamin-deficient cell was comparable to that in the control cell (**Supplementary Figure 2F**), suggesting that lamin knockdown had no affection on lipid oxidization. Therefore, these results indicate that lamin knockdown altered DNA repair choices and impaired mitochondrial activity.

### Lamin deficiency inhibits tumor growth by enhancing cytotoxic T-cell infiltration and activation

**Figure 3:**
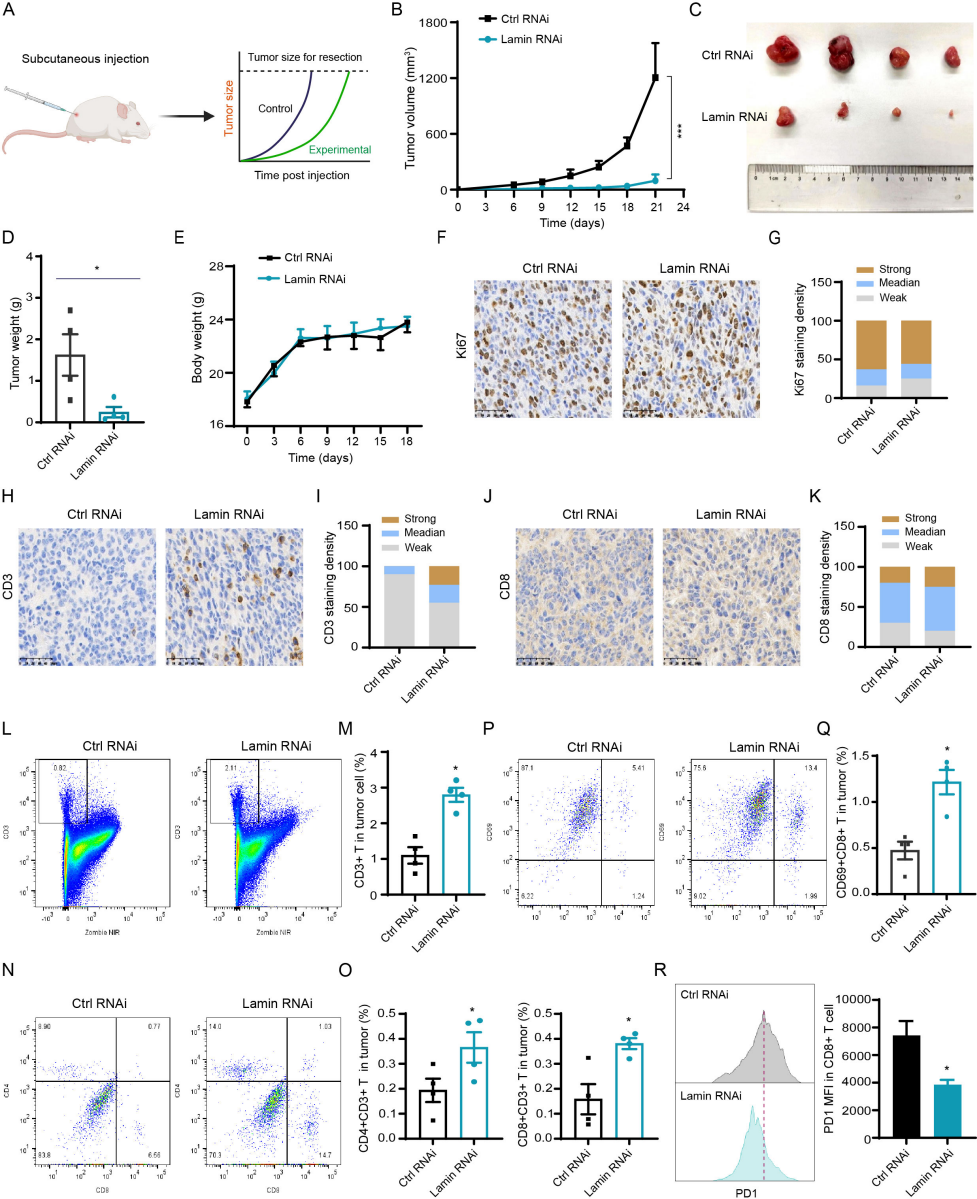
Lamin deficiency suppresses carcinogenesis via enhancing immune cell infiltration and activation. **(A)** Schematic diagram of mouse subcutaneous xenograft tumor model. **(B)** The growth curves of xenograft tumors without or with lamin knockdown in BALB/c mice. **(C)** Representative images of xenograft tumors without or with lamin knockdown. **(D)** Quantification of tumor weights as in **(C)**. **(E)** Body weight measurements of BALB/c mice carrying the control or lamin-deficient tumors. **(F)** IHC staining of Ki67 protein in tumors with or without lamin knockdown. Scale bar, 50 μm. **(G)** Quantification of Ki67 staining intensity in **(F)**. **(H)** IHC analysis of CD3 protein in tumors without or with lamin knockdown. Scale bar, 50 μm. **(I)** Quantification of CD3 staining intensity in **(H)**. **(J)** IHC staining of CD8 protein in tumors without or with lamin knockdown. Scale bar, 50 μm. **(K)** Quantification of CD8 staining intensity in **(J)**. **(L, M**) FACS analysis of CD3^+^ T cells in tumors with or without lamin knockdown. Representative FACS plots were shown in **(L)**, while the quantification of CD3^+^ T percentage was shown in **(M)**. **(N, O)** FACS analysis of CD4 and CD8 proteins in above CD3^+^ T cell population. Representative FACS plots were shown in **(N)**, while the quantifications of CD4^+^CD3^+^ and CD8^+^CD3^+^ T cell percentages were shown in **(O)**. **(P, Q)** FACS analysis of CD69 and CD8 proteins in tumors without or with lamin knockdown. Representative FACS plots of CD69 and CD8 proteins in CD3^+^ T cell population were shown in **(P)**, while the quantification of CD69^+^CD8^+^ T cell percentage in tumor cells was shown in **(Q)**. **(R)** FACS determination of PD1 protein level in CD8^+^ T cells from control or lamin-deficient tumors. Left: Representative FACS histograms; Right: Quantification of PD1 expression intensity in CD8^+^ T cells. Quantification is shown as mean ± SEM (n = 4). *P < 0.05, ***P< 0.001 versus the control RNAi.

To explore whether lamin knockdown in cancer stem-like cell affects tumorigenesis *in vivo*, we performed the subcutaneous tumor experiment in Balb/c mice (**Figure 3A**). In brief, cancer cells without or with lamin knockdown were infused into the right subcutaneous flank of 5-week-old mice, and their relevant tumors were monitored periodically. Lamin knockdown was shown to inhibit dramatically the growth of xenograft tumors (**Figure 3B**), resulting in the smaller volume and lower weight of lamin-deficient tumors (**Figures 3C, D**). The mice bearing either lamin-deficient or control tumors had gradually increased body weights (**Figure 3E**), suggesting their normal diet. There was no significant alteration in mouse spleen weights (**Supplementary Figure 3A**). Immunohistochemistry staining revealed that the Ki67 proliferation index was slightly lower in the lamin-deficient tumor (**Figure 3F, G**), indicating that the suppression of lamin knockdown on tumorigenesis may not be due to reduced cancer cell proliferation. Since the immune score is positively linked with patient prognosis, cancer patients with LMNA mutation were found to present higher infiltration score of immune cells such as cytotoxic CD8^+^ T and dendritic cells (**Supplementary Figures 3B, C**). Indeed, lamin-deficient tumors were shown to exhibit more CD3^+^ and CD8^+^ T lymphocytes than the control xenografts (**Figures 3H–K**). FACS analysis confirmed that the proportions of CD3^+^ T, CD4^+^ CD3^+^ T, and CD8^+^ CD3^+^ T lymphocytes were significantly higher in the lamin-deficient tumors (**Figures 3L–O**). Additionally, lamin-deficient tumors presented a much higher frequency of CD69^+^ CD3^+^ T cells than the control xenografts (**Figures 3P, Q**). These results suggest that lamin knockdown in cancer cells enhanced the infiltration and activation of cytotoxic T lymphocytes in the lamin-deficient tumor. Moreover, the expression level of immune checkpoint PD-1 on tumor-infiltrating CD8^+^ T cells was much lower in the lamin-deficient tumors than in the control xenografts (**Figure 3R**), suggesting the increased T-cell functionality in the context of lamin knockdown.

To test whether lamin knockdown impedes tumor growth mainly by enhancing T cell infiltration and activation, we conducted the subcutaneous xenograft tumor experiment in immunodeficient nude mice lacking T cells (**Supplementary Figure 3D**). Unexpectedly, the growth and weight of lamin-deficient tumors were comparable to that of the control xenografts in nude mice (**Supplementary Figures 3E, F**). The body weights of nude mice carrying either lamin-deficient or control tumors increased gradually over time (**Supplementary Figure 3G**). Thus, lamin knockdown within cancer cells could not inhibit tumor progression in T-cell-deficient nude mice. Moreover, we employed the *in vitro* co-culture system to measure if lamin knockdown in cancer cell affects other immune cells such as macrophage. The conditional medium of cancer cells was incubated with macrophage Raw264.7 cells for at least 48 hours. When compared to the control medium, the conditional media of lamin-deficient cancer cells did not change NLRP3 inflammasome and STING/TBK1 pathways in macrophage cells (**Supplementary Figure 4A**). The expressions of MHC molecule H-2Kb and co-stimulator factors (CD80 and CD86) on the surface of macrophages were not affected by the co-culture of lamin-deficient cancer cells (**Supplementary Figures 4B–D**), suggesting that lamin knockdown in cancer cell might not influence macrophage activation. Collectively, these data indicate that lamin knockdown within cancer stem-like cell restrained the *in vivo* tumorigenesis via boosting the infiltration and activity of tumor-infiltrating T cells.

### Lamin knockdown modulates cancer cell stemness and immunity via the HIF signaling

**Figure 4:**
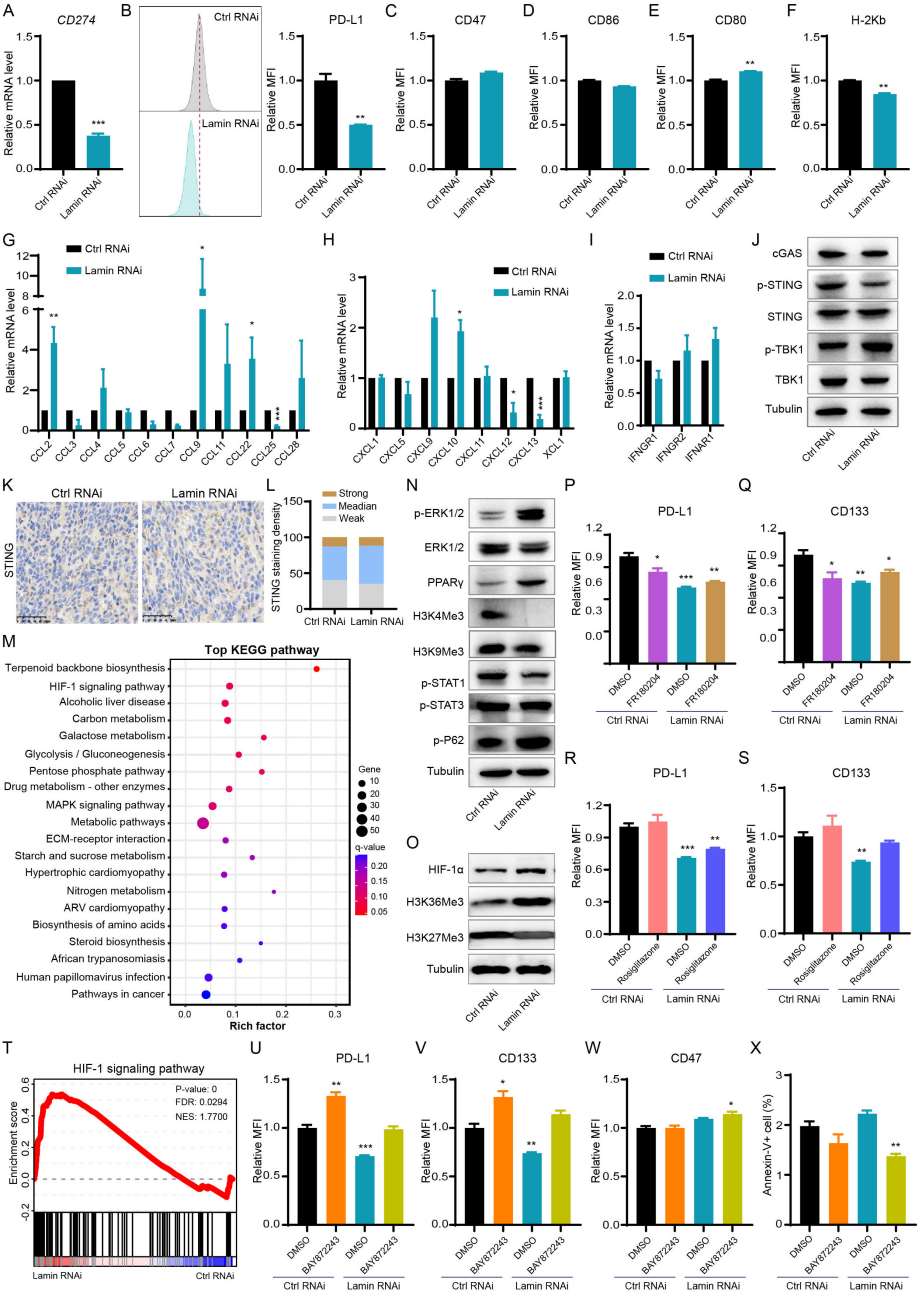
Lamin deficiency regulates cancer-cell-intrinsic immunity and HIF-1 signaling. **(A)** Q-PCR analysis of *CD274* mRNA level in cancer cells without or with lamin knockdown. **(B)** FACS analysis of PD-L1 protein level in the control and lamin-deficient cancer cells. Left: Representative FACS histograms; Right: Relative fold change of PD-L1 expression intensity. **(C)** FACS analysis of CD47 expression level on cancer cells without or with lamin knockdown. **(D)** FACS determination of CD86 protein level on the control and lamin-deficient cells. **(E)** FACS analysis of CD80 expression level on cancer cells without or with lamin deficiency. **(F)** FACS determination of H-2Kb protein level on the control and lamin-deficient cancer cells. **(G)** Q-PCR analyses of cytokine levels in cancer cells without or with lamin deficiency. **(H)** Q-PCR analyses of chemokine expressions in the control and lamin-deficient cancer cells. **(I)** Q-PCR analyses of IFN-γ receptors in cancer cells without or with lamin deficiency. **(J)** Western blotting analysis of cGAS-STING pathway proteins in cancer cells without or with lamin deficiency. **(K)** IHC staining of STING protein in tumors with or without lamin knockdown. Scale bar, 50 μm. **(L)** Quantification of STING staining intensity in **(K)**. **(M)** KEGG pathway analysis of differentially expressed genes in the RNA-seq data of control and lamin-deficient cancer cells. **(N)** Western blotting analysis of histone methylation and signaling molecules in cancer cells without or with lamin deficiency. **(O)** Western blotting analysis of histone methylation and HIF-1α molecules in cancer cells without or with lamin deficiency. **(P)** FACS analysis of PD-L1 expression level on cancer cells with or without FR180204 treatment. **(Q)** FACS analysis of CD133 expression change in the control and lamin-deficient cells with or without FR180204 treatment. **(R)** FACS determination of PD-L1 protein level on the control and lamin-deficient cells without or with rosiglitazone treatment. **(S)** FACS analysis of CD133 expression change in the control and lamin-deficient cells with DMSO or rosiglitazone treatment. **(T)** GSEA analysis of HIF-1 signaling pathway in the RNA-seq data of control and lamin-deficient cancer cells. **(U)** FACS analysis of PD-L1 protein level on the control and lamin-deficient cells treated with DMSO or BAY872243. **(V)** FACS analysis of CD133 expression change in the control and lamin-deficient cells receiving DMSO or BAY872243 treatment. **(W)** FACS analysis of CD47 level on the control and lamin-deficient cells treated with DMSO or BAY872243. **(X)** FACS analysis of apoptotic cell percentage in control and lamin-deficient cells receiving DMSO or BAY872243 treatment. Quantification is shown as mean ± SEM (n = 3). *P < 0.05, **P < 0.01, ***P< 0.001 versus the control RNAi or DMSO-treated control RNAi.

Given the promotion of lamin-deficient cancer cells on T-cell activation and infiltration, we next tried to assess whether lamin knockdown regulates immune checkpoint dynamic in cancer cell. The PD-L1 on cancer cell is a well-known immune checkpoint molecule impairing antitumor immunity via interaction with PD1 on T cell (34). The *CD274* mRNA encoding PD-L1 protein was significantly reduced by lamin knockdown (**Figure 4A**). FACS analysis confirmed that lamin knockdown led to a notable reduction in PD-L1 protein expression on cancer cells (**Figure 4B**). Conversely, the expression levels of CD47 and CD86 proteins on cancer cells were not affected by lamin knockdown (**Figures 4C, D**, **Supplementary Figure 5A**). CD47 is a surface receptor on cancer cell and delivers the “don’t eat me” signal by interacting with SIRPα on myeloid cells (35), while costimulatory molecules CD80 and CD86 participate in the regulation of antigen presentation and T cell activation (36, 37). There was a slight alteration in CD80 and H-2Kb expression after lamin knockdown (**Figures 4E, F**, **Supplementary Figures 5B, C**), suggesting their minimal contribution to the anti-tumor effect of lamin knockdown. H-2Kb is a classical MHC-I molecule presenting tumor antigen peptides to cytotoxic T cell (38). Interestingly, lamin-deficient cancer cells were found to present higher expression levels of many inflammatory chemokines such as *CCL2*, *CCL9*, *CCL22*, *CXCL9*, and *CXCL10* (**Figures 4G, H**). Lamin knockdown seemed not to affect the IFNGR2 and IFNAR1 levels (**Figure 4I**). IFNGR1/2 and IFNAR1, serving as the receptor for IFN-γ and IFN-α respectively, are critical in cancer cell fate and tumor microenvironment (39, 40). Given the importance of cGAS-STING pathway in immune checkpoint signaling (28, 41), we then examined how lamin knockdown impacts the cGAS-STING pathway. The transcriptional and protein levels of cGAS, STING, and TBK1 were not changed by lamin knockdown (**Figure 4J**, **Supplementary Figure 5D**). Immunohistochemistry analysis revealed that lamin-deficient tumor had a comparable STING intensity than the control xenograft (**Figures 4K, L**), indicating that the STING/TBK1 pathway may not be the downstream mechanism. Remarkably, bioinformatic analysis showed that lamin knockdown could regulate the expressions of many functional genes enriched in AMPK, MAPK, and HIF-1 signaling pathways (**Figure 4M**, **Supplementary Figure 5E**). Indeed, lamin knockdown gave rise to the downregulation of STAT1 phosphorylation and the upregulation of ERK phosphorylation and PPARγ (**Figure 4N**). Both HIF-1α and H3K36Me3 levels were also increased by the lamin knockdown (**Figure 4O**). To determine which pathway is the major one, we treated the control and lamin-deficient cancer cells with pathway inhibitor or activator. Both ERK inhibitor FR180204 and PPARγ agonist Rosiglitazone were found to generate a slight restoration on the lower levels of PD-L1 and CD133 caused by lamin knockdown (**Figures 4P–S**). However, neither ERK inhibitor nor PPARγ agonist could influence CD47 expression in lamin-deficient cancer cells (**Supplementary Figures 5F, G**). Unexpectedly, the selective TBK1 inhibitor GSK8612 reduced the PD-L1, CD133, and CD47 expressions in the absence or presence of lamin knockdown (**Supplementary Figures 5H–J**). Consistent with the promotion of lamin knockdown on HIF-1 signaling (**Figures 4O, T**), the HIF-1 inhibitor BAY872243 was shown to rescue the suppression of lamin knockdown on immune checkpoint PD-L1 and stemness marker CD133 (**Figures 4U, V**). The BAY872243 treatment generated an enhancement of CD47 level on lamin-deficient cell rather than the control cell (**Figure 4W**). The BAY872243 treatment led to the reduction of cellular apoptosis (**Figure 4X**), indicating the safety of its application dosage. Moreover, the BAY872243 was found to restore slightly the inhibition of lamin knockdown on cell cycle progression, as evidenced partially by the increase of G2/M phases in lamin-deficient cells with BAY872243 treatment (**Supplementary Figures 5K–M**). Overall, these data suggest that lamin knockdown modulated cancer cell stemness and inflammatory factors through the HIF-1 pathway.

### Lamin-deficient cancer cells are more sensitive to chemotherapy

**Figure 5:**
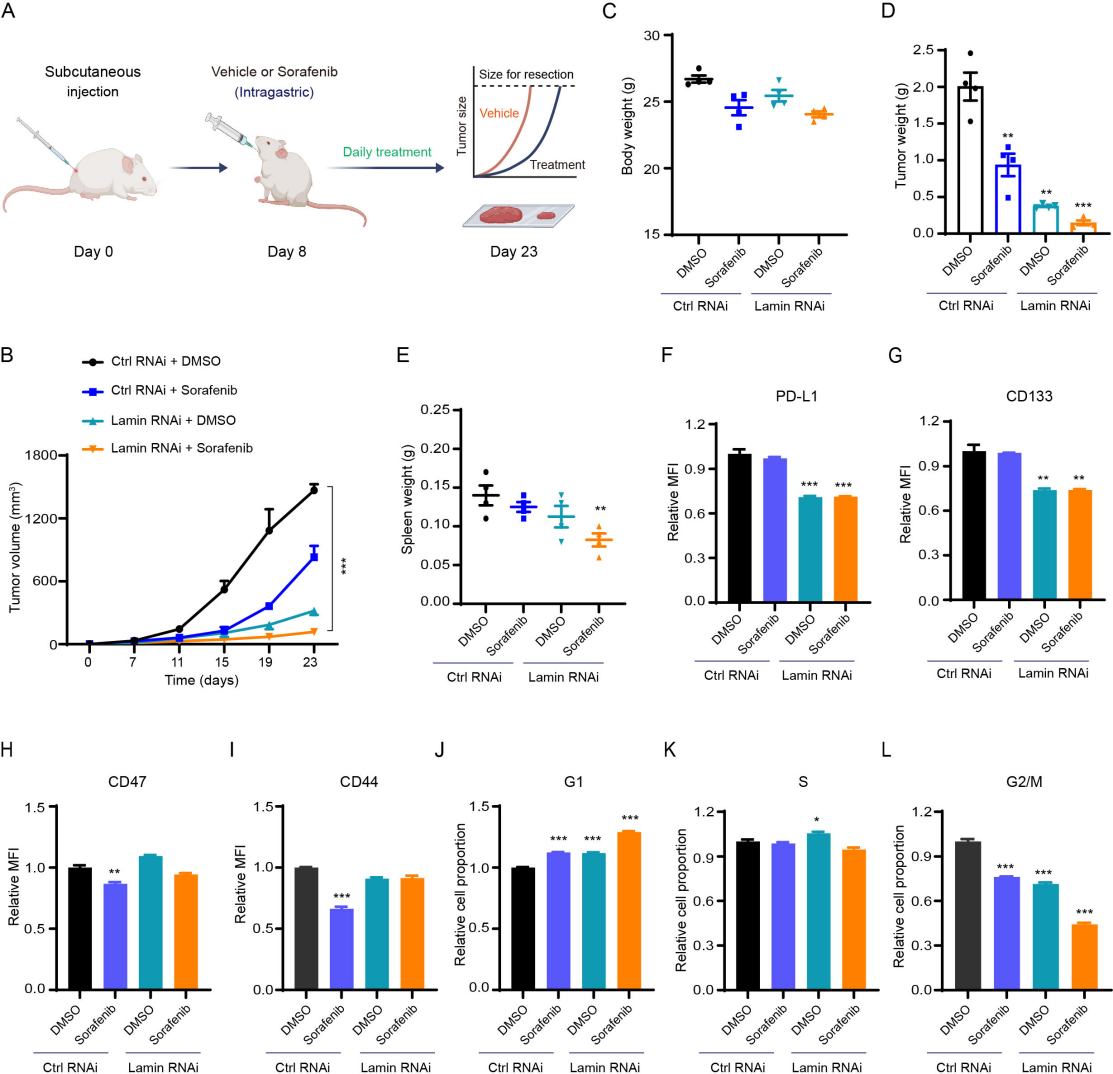
Lamin deficiency enhances chemosensitivity. **(A)** Schematic illustration of sorafenib application in the mouse tumor model. **(B)** The growth curves of control and lamin-deficient xenograft tumors in BALB/c mice receiving DMSO or sorafenib treatment. **(C)** Body weights of BALB/c mice bearing the control or lamin-deficient allografts at the experimental endpoint. **(D)** The weights of control and lamin-deficient xenograft tumors from BALB/c mice treated with DMSO or sorafenib. **(E)** The spleen weights of BALB/c mice treated as **(D)**. **(F)** FACS analysis of PD-L1 expression change in the control and lamin-deficient cancer cells receiving DMSO or sorafenib treatment. **(G)** FACS analysis of CD133 expression level on the control and lamin-deficient cells treated with DMSO or sorafenib. **(H)** FACS analysis of CD47 expression change in the control and lamin-deficient cell with or without sorafenib treatment. **(I)** FACS analysis of CD44 expression on the control and lamin-deficient cell treated with DMSO or sorafenib. **(J-L)** Relative fold changes of the percentage of G1 **(J)**, S **(K)**, and G2/M **(L)** in the control and lamin-deficient cells treated with DMSO or sorafenib. Quantification is shown as mean ± SEM (n = 4). *P < 0.05, **P < 0.01, ***P< 0.001 versus the DMSO-treated control RNAi.

To evaluate the responsivity of lamin-deficient cancer cells to anti-tumor drugs, we injected the immunocompetent mice with cancer cells and then administrated the chemotherapeutic agent sorafenib periodically (**Figure 5A**). Sorafenib, a multiple-target tyrosine kinase inhibitor, has been used clinically for the treatment of many solid tumors such as hepatic cancer through inhibiting cancer cell proliferation and inducing apoptosis (42, 43). The sorafenib inhibited xenograft tumor growth in comparison to the vehicle treatment (**Figure 5B**). Notably, the inhibition extent of lamin knockdown on tumor growth was much stronger than the sorafenib application (**Figure 5B**, **Supplementary Figure 5N**). The Balb/c mice receiving sorafenib treatment displayed slightly lower body weights at the experimental endpoint (**Figure 5C**). The sorafenib administration was found to generate smaller tumor volume and weight even in the presence of lamin knockdown (**Figures 5B–D**), presenting the enhanced sensitivity of lamin-deficient cancer cells to the chemotherapeutic agent. The spleen weights from different groups of mice were comparable to each other, except for the mice with lamin-deficient tumor and sorafenib treatment (**Figure 5E**). Although lamin-deficient cancer cells had lower PD-L1 and stemness markers (**Figures 1C**, **4B**), the sorafenib did not alter PD-L1 and CD133 levels on cancer cells with or without lamin knockdown (**Figures 5F, G**). Unlike the lamin knockdown, the sorafenib led to a decrease in CD47 expression (**Figure 5H**). The level of CD44 protein in the control cell, rather than in lamin-deficient cell, was reduced by the sorafenib (**Figure 5I**). Moreover, the sorafenib increased the percentage of the G1 phase and decreased the proportion of the G2/M stages (**Figures 5J–L**), which is similar to the lamin knockdown. The combination of lamin knockdown and sorafenib could induce the highest proportion of G1 phase and generate the lowest percentage of G2/M phases (**Figures 5J–L**). These findings indicate that lamin knockdown in cancer cells enhanced their sensitivity to chemotherapy.

### Lamin deficiency in breast cancer cell suppresses PD-L1 expression and subcutaneous tumor growth

To determine whether all lamin knockdown changes PD-L1 expression and tumorigenesis in another cell model, we engineered the three lamin subtypes in murine breast cancer cell line using above shRNA-mediated knockdown system. The transcript levels of *LMNA*, *LMNB1*, and *LMNB2* were dramatically reduced by the lamin knockdown (**Supplementary Figure 6A**). FACS analysis revealed that lamin-deficient breast cancer cells had much lower PD-L1 protein level and intracellular ROS amount (**Supplementary Figures 6B, C**). After subcutaneous injection into the immunocompetent Balb/c mice, lamin-deficient breast cancer cell was found to grow slowly (**Supplementary Figure 6D**). Consequently, the volume and weight of lamin-deficient breast tumors were much lower than the control xenografts (**Supplementary Figures 6E, F**). There existed no alteration in mouse spleen weights (**Supplementary Figure 6G**). Thus, these data suggest that lamin knockdown also impeded PD-L1 expression and subcutaneous tumor progression in second cancer cell model.

## Discussion

Cancer stem-like cells are often quiescent and possess the remarkable capacity to rebuild tumor mass *in situ* or in a new place, contributing to their resistance against chemotherapy, radiotherapy, and immunotherapy (23, 24). Herein, we demonstrated that nuclear lamin is critical in maintaining cancer cell stemness and proliferation rather than the survival. Lamin-deficient cancer cells were shown to present lower stemness, impaired mitochondrial activity, and diminished tumorigenicity. The high plasticity of cancer cells enables them to adapt and survive under diverse conditions. The disruption of cancer cell plasticity by lamin knockdown may be owing to the nuclear deformation, genome instability, chromatin abnormality, and/or dysfunctional nuclear-cytoplasmic interaction. Lamin deficiency is likely insufficient to provide mechanical support and architectural organization for the nucleus and chromatin, leading to the aberrant expression profiles of functional genes involved in diverse cellular processes such as genomic damage repair and metabolic homeostasis. Indeed, lamin-deficient cells exhibited irregular and flexural nuclear morphology, suggesting the impairment of nuclear transcription. In this scenario, mitochondria might become dysfunctional since most mitochondrial proteins are encoded by nuclear DNA. The reduction of mitochondrial proteins and activity by lamin knockdown suggests that lamin-deficient cells may not generate enough metabolites and energy to sustain their stemness network and cell-cycle transition. It is of considerable interest to further elucidate the contribution of nuclear gene dysregulation in abnormal cell behaviors. Upon the importance of lamin proteins in nucleus-cytoskeleton connection (44, 45), lamin deficiency has the chance to alter cellular size. As thus, the detection of spontaneous lamin abnormality by the cell population in a specific tumor could serve as a new prognostic factor.

The specific expression and mutation profiles of lamin proteins have been indicated to act as valuable markers for predicting cancer prognosis and tailoring potential treatments (46–49). Our study shows evidence that the knockdown of lamin within cancer stem-like cell gave rise to a significant reduction in tumor growth and weight. Hence, targeting lamin expression or function could be viable to disrupt the plasticity of cancer stem-like cells and potentially improve the efficiency of cancer therapy. Supporting this notion, lamin knockdown was observed to enhance the chemosensitivity of cancer cells, as demonstrated by the smallest tumor volume and weight in the combination of chemotherapy and lamin deficiency. Since the chemotherapy could not alter the immune checkpoints and stemness molecules in cancer stem-like cell, lamin deficiency may influence the expression of drug-resistance genes to promote the therapeutic efficacy of chemotherapy. It is important to note that cancer cells within tumor mass encounter various stressors including but not limited to the DNA damage and immune cell attacks (50, 51). While lamin-deficient tumors had a slight reduction in the cell proliferative rate, they exhibited a dramatic increase in T-cell infiltration and activation. In this context, the reduction in tumor size by lamin knockdown largely relies on the host’s immune system rather than direct effects on the tumor cells. Consistently, lamin knockdown within cancer stem-like cells did not impact tumor progression in the immunodeficient nude mice. These results highlight the role of nuclear lamin in regulating cancer cell niche, which is essential for stem-like cell maintenance and tumorigenesis. The tumor microenvironment is an orchestral network comprising the immunocytes, endothelial cells, fibroblasts, pericytes, and extracellular matrix (50, 52). Further studies are necessary to investigate how lamin knockdown in cancer stem-like cell modulates extracellular factors and other cell types beyond T lymphocytes. Chemokines within the tumor microenvironment play crucial roles in guiding immune cell migration and connections with cancer cell (53, 54). Lamin-deficient cancer cells were demonstrated to exhibit significantly higher levels of several chemokine genes such as *CCL2* and *CXCL10*. More researches are needed to determine which secretory chemokine primarily facilitates the infiltration of T cells into lamin-deficient tumors. Another notable point is that lamin-deficient cancer cells exhibited much lower immunoinhibitory PD-L1 expression at both transcriptional and protein levels, which may be the major contributor to the enhanced T cell activation in lamin-deficient tumors. Therefore, the disruption of lamin function is reasonable to enhance the immunogenicity of cancer cells and improve the efficacy of immunotherapy. It would be intriguing to explore whether the knockdown of nuclear lamin can synergize with current immunotherapies to counteract stem-like cell behavior and impede tumor growth.

Beyond its implications in cancer cell plasticity, lamin knockdown herein was indicated to suppress the transition of stem cells to hematopoietic cell lineage and sperm cell feature (**Supplementary Figures 6A, B**). Consistently, lamin-deficient cancer stem-like cells had much lower levels of hematopoietic factors such as *c-myb* and *T* (**Supplementary Figure 6C**). This phenomenon may be due to the relatively conventional signaling network that governs the stemness/differentiation switch among distinct stem cell, as evidenced by the reductive expression of stemness-related ALDH1 and NANOG in cancer stem-like cell (**Figure 1D**) and OCT4 in pluripotent stem cell (55). Proper nuclear architecture in hematopoietic progenitors is essential for preserving their stem cell property and function. Lamin knockdown may lead to the expression abnormality of functional genes critical for stem cell maintenance and lineage commitment through regulating chromatin accessibility and/or its interaction with transcriptional factors. Future research is necessary to unravel molecular mechanisms by which the lamin proteins govern the fate decision of primitive hematopoietic cell. It will also be interesting to explore how lamin mutation in normal stem cells predicts the onset of adult diseases such as genetic anemia.

## Data Availability

The original contributions presented in the study can be found in the article/**Supplementary Material**. Further inquiries could be directed to the corresponding author.

## References

[1] de Leeuw R Gruenbaum Y Medalia O. Nuclear lamins: thin filaments with major functions. Trends Cell Biol. (2018) 28:34. doi: 10.1016/j.tcb.2017.08.004, PMID: 28893461

[2] Gruenbaum Y Medalia O. Lamins: the structure and protein complexes. Curr Opin Cell Biol. (2015) 32:7. doi: 10.1016/j.ceb.2014.09.009, PMID: 25460776

[3] van Steensel B Belmont AS. Lamina-associated domains: links with chromosome architecture, heterochromatin, and gene repression. Cell. (2017) 169:780. doi: 10.1016/j.cell.2017.04.022, PMID: 28525751 PMC5532494

[4] Briand N Collas P. Lamina-associated domains: peripheral matters and internal affairs. Genome Biol. (2020) 21:85. doi: 10.1186/s13059-020-02003-5, PMID: 32241294 PMC7114793

[5] Shimamoto Y Tamura S Masumoto H Maeshima K. Nucleosome-nucleosome interactions via histone tails and linker DNA regulate nuclear rigidity. Mol Biol Cell. (2017) 28:1580. doi: 10.1091/mbc.e16-11-0783, PMID: 28428255 PMC5449155

[6] Kovacs MT Vallette M Wiertsema P Dingli F Loew D Nader GPF. DNA damage induces nuclear envelope rupture through ATR-mediated phosphorylation of lamin A/C. Mol Cell. (2023) 83:3659. doi: 10.1016/j.molcel.2023.09.023, PMID: 37832547 PMC10597398

[7] Dong CH Jiang T Yin H Song H Zhang Y Geng H. LMNB2 promotes the progression of colorectal cancer by silencing p21 expression. Cell Death Dis. (2021) 12:331. doi: 10.1038/s41419-021-03602-1, PMID: 33782407 PMC8007612

[8] Wang Y Chen Q Wu D Chen Q Gong G He L. Lamin-A interacting protein Hsp90 is required for DNA damage repair and chemoresistance of ovarian cancer cells. Cell Death Dis. (2021) 12:786. doi: 10.1038/s41419-021-04074-z, PMID: 34381017 PMC8358027

[9] Jia Y Vong JS Asafova A Garvalov BK Caputo L Cordero J. Lamin B1 loss promotes lung cancer development and metastasis by epigenetic derepression of RET. J Exp Med. (2019) 216:1377. doi: 10.1084/jem.20181394, PMID: 31015297 PMC6547854

[10] Liu H Li D Zhou L Kan S He G Zhou K. LMNA functions as an oncogene in hepatocellular carcinoma by regulating the proliferation and migration ability. J Cell Mol Med. (2020) 24:12008. doi: 10.1111/jcmm.15829, PMID: 32896989 PMC7578910

[11] Kirkland NJ Skalak SH Whitehead AJ Hocker JD Beri P Vogler G. Age-dependent Lamin changes induce cardiac dysfunction via dysregulation of cardiac transcriptional programs. Nat Aging. (2023) 3:17. doi: 10.1038/s43587-022-00323-8, PMID: 36845078 PMC9956937

[12] Hasselberg NE Haland TF Saberniak J Brekke PH Berge KE Leren TP. Lamin A/C cardiomyopathy: young onset, high penetrance, and frequent need for heart transplantation. Eur Heart J. (2018) 39:853. doi: 10.1093/eurheartj/ehx596, PMID: 29095976 PMC5939624

[13] de Toledo M Lopez-Mejia IC Cavelier P Pratlong M BarraChina C Gromada X. Lamin C counteracts glucose intolerance in aging, obesity, and diabetes through β-cell adaptation. Diabetes. (2020) 69:647. doi: 10.2337/db19-0377, PMID: 32005707

[14] Luo F Han J Chen Y Yang K Zhang Z Li J. Lamin B1 promotes tumor progression and metastasis in primary prostate cancer patients. Future Oncol. (2021) 17:663. doi: 10.2217/fon-2020-0825, PMID: 33112662

[15] Li L Du Y Kong X Li Z Jia Z Cui J. Lamin B1 is a novel therapeutic target of betulinic acid in pancreatic cancer. Clin Cancer Res. (2013) 19:4651. doi: 10.1158/1078-0432.CCR-12-3630, PMID: 23857605 PMC3800003

[16] Yang Y Xiao W Liu R Gao L Chen J Kan H. A lamin family-based signature predicts prognosis and immunotherapy response in hepatocellular carcinoma. J Immunol Res. (2022) 2022:4983532. doi: 10.1155/2022/4983532, PMID: 36405011 PMC9673181

[17] Xin H Tang Y Jin YH Li HL Tian Y Yu C. Knockdown of LMNA inhibits Akt/β-catenin-mediated cell invasion and migration in clear cell renal cell carcinoma cells. Cell Adh Migr. (2023) 17:1. doi: 10.1080/19336918.2023.2260644, PMID: 37749865 PMC10524799

[18] Kong L Schäfer G Bu H Zhang Y Zhang Y Klocker H. Lamin A/C protein is overexpressed in tissue-invading prostate cancer and promotes prostate cancer cell growth, migration and invasion through the PI3K/AKT/PTEN pathway. Carcinogenesis. (2012) 33:751. doi: 10.1093/carcin/bgs022, PMID: 22301279

[19] Huang Y Zhang L Liu T Liang E. LMNB1 targets FOXD1 to promote progression of prostate cancer. Exp Ther Med. (2023) 26:513. doi: 10.3892/etm.2023.12212, PMID: 37840569 PMC10570766

[20] Ji J Li H Chen J Wang W. Lamin B2 contributes to the proliferation of bladder cancer cells via activating the expression of cell division cycle−associated protein 3. Int J Mol Med. (2022) 50:111. doi: 10.3892/ijmm.2022.5168, PMID: 35775376 PMC9282643

[21] Wang J Ma M Liu X. Lamins B2 promotes esophageal cancer by stimulating proliferation and inhibiting apoptosis. Ann Clin Lab Sci. (2022) 52:202., PMID: 35414499

[22] Chu X Tian W Ning J Xiao G Zhou Y Wang Z. Cancer stem cells: advances in knowledge and implications for cancer therapy. Signal Transduct Target Ther. (2024) 9:170. doi: 10.1038/s41392-024-01851-y, PMID: 38965243 PMC11224386

[23] Loh JJ Ma S. Hallmarks of cancer stemness. Cell Stem Cell. (2024) 31:617. doi: 10.1016/j.stem.2024.04.004, PMID: 38701757

[24] Saw PE Liu Q Wong PP Song E. Cancer stem cell mimicry for immune evasion and therapeutic resistance. Cell Stem Cell. (2024) 31:1101. doi: 10.1016/j.stem.2024.06.003, PMID: 38925125

[25] Xu C Jin G Wu H Cui W Wang YH Manne RK. SIRPγ-expressing cancer stem-like cells promote immune escape of lung cancer via Hippo signaling. J Clin Invest. (2022) 132:e141797. doi: 10.1172/JCI141797, PMID: 35229723 PMC8884909

[26] Tsunekuni K Konno M Haraguchi N Koseki J Asai A Matsuoka K. CD44/CD133-positive colorectal cancer stem cells are sensitive to trifluridine exposure. Sci Rep. (2019) 9:14861. doi: 10.1038/s41598-019-50968-6, PMID: 31619711 PMC6795793

[27] Wang X Chai Y Quan Y Wang J Song J Zhou W. NPM1 inhibits tumoral antigen presentation to promote immune evasion and tumor progression. J Hematol Oncol. (2024) 17:97. doi: 10.1186/s13045-024-01618-6, PMID: 39402629 PMC11479574

[28] Tang M Yin S Zeng H Huang A Huang Y Hu Z. The P286R mutation of DNA polymerase ϵ activates cancer-cell-intrinsic immunity and suppresses endometrial tumorigenesis via the cGAS-STING pathway. Cell Death Dis. (2024) 15:69. doi: 10.1038/s41419-023-06418-3, PMID: 38238314 PMC10796917

[29] Zhou Y Peng X Fang C Peng X Tang J Wang Z. Histones methyltransferase NSD3 inhibits lung adenocarcinoma glycolysis through interacting with PPP1CB to decrease STAT3 signaling pathway. Adv Sci (Weinh). (2024) 11:e2400381. doi: 10.1002/advs.202400381, PMID: 39119928 PMC11481231

[30] Liu Y Wang H. Biomarkers and targeted therapy for cancer stem cells. Trends Pharmacol Sci. (2024) 45:56. doi: 10.1016/j.tips.2023.11.006, PMID: 38071088 PMC10842814

[31] Chen Y Qiang Y Fan J Zheng Q Yan L Fan G. Aggresome formation promotes ASK1/JNK signaling activation and stemness maintenance in ovarian cancer. Nat Commun. (2024) 15:1321. doi: 10.1038/s41467-024-45698-x, PMID: 38351029 PMC10864366

[32] Kuhar R Gwiazda KS Humbert O Mandt T Pangallo J Brault M. Novel fluorescent genome editing reporters for monitoring DNA repair pathway utilization at endonuclease-induced breaks. Nucleic Acids Res. (2014) 42:e4. doi: 10.1093/nar/gkt872, PMID: 24121685 PMC3874187

[33] Nagel ZD Margulies CM Chaim IA McRee SK Mazzucato P Ahmad A. Multiplexed DNA repair assays for multiple lesions and multiple doses via transcription inhibition and transcriptional mutagenesis. Proc Natl Acad Sci U S A. (2014) 111:E1823. doi: 10.1073/pnas.1401182111, PMID: 24757057 PMC4020053

[34] Kornepati AVR Vadlamudi RK Curiel TJ. Programmed death ligand 1 signals in cancer cells. Nat Rev Cancer. (2022) 22:174. doi: 10.1038/s41568-021-00431-4, PMID: 35031777 PMC9989967

[35] Osorio JC Smith P Knorr DA Ravetch JV. The antitumor activities of anti-CD47 antibodies require Fc-FcγR interactions. Cancer Cell. (2023) 41:2051. doi: 10.1016/j.ccell.2023.10.007, PMID: 37977147 PMC10842210

[36] Vackova J Polakova I Johari SD Smahel M. CD80 expression on tumor cells alters tumor microenvironment and efficacy of cancer immunotherapy by CTLA-4 blockade. Cancers (Basel). (2021) 13:1935. doi: 10.3390/cancers13081935, PMID: 33923750 PMC8072777

[37] Kennedy A Waters E Rowshanravan B Hinze C Williams C Janman D. Differences in CD80 and CD86 transendocytosis reveal CD86 as a key target for CTLA-4 immune regulation. Nat Immunol. (2022) 23:1365. doi: 10.1038/s41590-022-01289-w, PMID: 35999394 PMC9477731

[38] Cornel AM Mimpen IL Nierkens S. MHC class I downregulation in cancer: underlying mechanisms and potential targets for cancer immunotherapy. Cancers (Basel). (2020) 12:1760. doi: 10.3390/cancers12071760, PMID: 32630675 PMC7409324

[39] Gocher AM Workman CJ Vignali DAA. Interferon-γ: teammate or opponent in the tumour microenvironment? Nat Rev Immunol. (2022) 22:158. doi: 10.1038/s41577-021-00566-3, PMID: 34155388 PMC8688586

[40] Holicek P Guilbaud E Klapp V Truxova I Spisek R Galluzzi L. Type I interferon and cancer. Immunol Rev. (2024) 321:115. doi: 10.1111/imr.13272, PMID: 37667466

[41] Zhu W Rao J Zhang LH Xue KM Li L Li JJ. OMA1 competitively binds to HSPA9 to promote mitophagy and activate the cGAS-STING pathway to mediate GBM immune escape. J Immunother Cancer. (2024) 12:e008718. doi: 10.1136/jitc-2023-008718, PMID: 38604814 PMC11015223

[42] Tsui YM Chan LK Ng IO. Cancer stemness in hepatocellular carcinoma: mechanisms and translational potential. Br J Cancer. (2020) 122:1428. doi: 10.1038/s41416-020-0823-9, PMID: 32231294 PMC7217836

[43] Wei JC Meng FD Qu K Wang ZX Wu QF Zhang LQ. Sorafenib inhibits proliferation and invasion of human hepatocellular carcinoma cells via up-regulation of p53 and suppressing FoxM1. Acta Pharmacol Sin. (2015) 36:241. doi: 10.1038/aps.2014.122, PMID: 25557114 PMC4326788

[44] Vahabikashi A Adam SA Medalia O Goldman RD. Nuclear lamins: Structure and function in mechanobiology. APL Bioeng. (2022) 6:011503. doi: 10.1063/5.0082656, PMID: 35146235 PMC8810204

[45] Skory RM Moverley AA Ardestani G Alvarez Y Domingo-Muelas A Pomp O. The nuclear lamina couples mechanical forces to cell fate in the preimplantation embryo via actin organization. Nat Commun. (2023) 14:3101. doi: 10.1038/s41467-023-38770-5, PMID: 37248263 PMC10226985

[46] Pei S Wang X Wang X Huang H Tao H Xie B. Aberrant nuclear lamina contributes to the Malignancy of human gliomas. J Genet Genomics. (2022) 49:132. doi: 10.1016/j.jgg.2021.08.013, PMID: 34530169

[47] Izdebska M Gagat M Grzanka A. Overexpression of lamin B1 induces mitotic catastrophe in colon cancer LoVo cells and is associated with worse clinical outcomes. Int J Oncol. (2018) 52:89. doi: 10.3892/ijo.2017.4182, PMID: 29115590 PMC5743383

[48] Willis ND Cox TR Rahman-Casañs SF Smits K Przyborski SA van den Brandt P. Lamin A/C is a risk biomarker in colorectal cancer. PloS One. (2008) 3:e2988. doi: 10.1371/journal.pone.0002988, PMID: 18714339 PMC2496895

[49] Kong W Wu Z Yang M Zuo X Yin G Chen W. LMNB2 is a prognostic biomarker and correlated with immune infiltrates in hepatocellular carcinoma. IUBMB Life. (2020) 72:2672. doi: 10.1002/iub.2408, PMID: 33211382

[50] de Visser KE Joyce JA. The evolving tumor microenvironment: From cancer initiation to metastatic outgrowth. Cancer Cell. (2023) 41:374. doi: 10.1016/j.ccell.2023.02.016, PMID: 36917948

[51] Jin MZ Jin WL. The updated landscape of tumor microenvironment and drug repurposing. Signal Transduct Target Ther. (2020) 5:166. doi: 10.1038/s41392-020-00280-x, PMID: 32843638 PMC7447642

[52] Anderson NM Simon MC. The tumor microenvironment. Curr Biol. (2020) 30:R921. doi: 10.1016/j.cub.2020.06.081, PMID: 32810447 PMC8194051

[53] Dong C. Cytokine regulation and function in T cells. Annu Rev Immunol. (2021) 39:51. doi: 10.1146/annurev-immunol-061020-053702, PMID: 33428453

[54] Kohli K Pillarisetty VG Kim TS. Key chemokines direct migration of immune cells in solid tumors. Cancer Gene Ther. (2022) 29:10. doi: 10.1038/s41417-021-00303-x, PMID: 33603130 PMC8761573

[55] Wang Y Elsherbiny A Kessler L Cordero J Shi H Serke H. Lamin A/C-dependent chromatin architecture safeguards naïve pluripotency to prevent aberrant cardiovascular cell fate and function. Nat Commun. (2022) 13:6663. doi: 10.1038/s41467-022-34366-7, PMID: 36333314 PMC9636150

