# Supplementary material for: Lamins regulate cancer cell plasticity and chemosensitivity
Source: Front Oncol. 2025 Jul 10;15:1599175. doi: 10.3389/fonc.2025.1599175 (PMC12286797; doi:10.3389/fonc.2025.1599175)
Supplement: Supplementary file 1 [file DataSheet1.docx]

**Supplementary materials for this article are as follows:**

Figure S1. Lamin transcripts are abnormally expressed in many human tumors.

Figure S2. Lamin knockdown alters nuclear shape and some cellular processes.

Figure S3. Lamin knockdown does not impair tumor growth in nude mice.

Figure S4. Lamin knockdown in cancer cell does not activate macrophage.

Figure S5. Both PPAR-γ agonist and TBK1 inhibitor are unable to rescue the effects of lamin knockdown.

Figure S6. Lamin knockdown in mammary cancer cell inhibits PD-L1 expression, ROS generation, and subcutaneous tumor growth.

Figure S7. All lamin knockdown likely inhibits hematopoietic differentiation.

Table S1. The list of shRNA sequences.

Table S2. The sequences of RT-PCR primers.


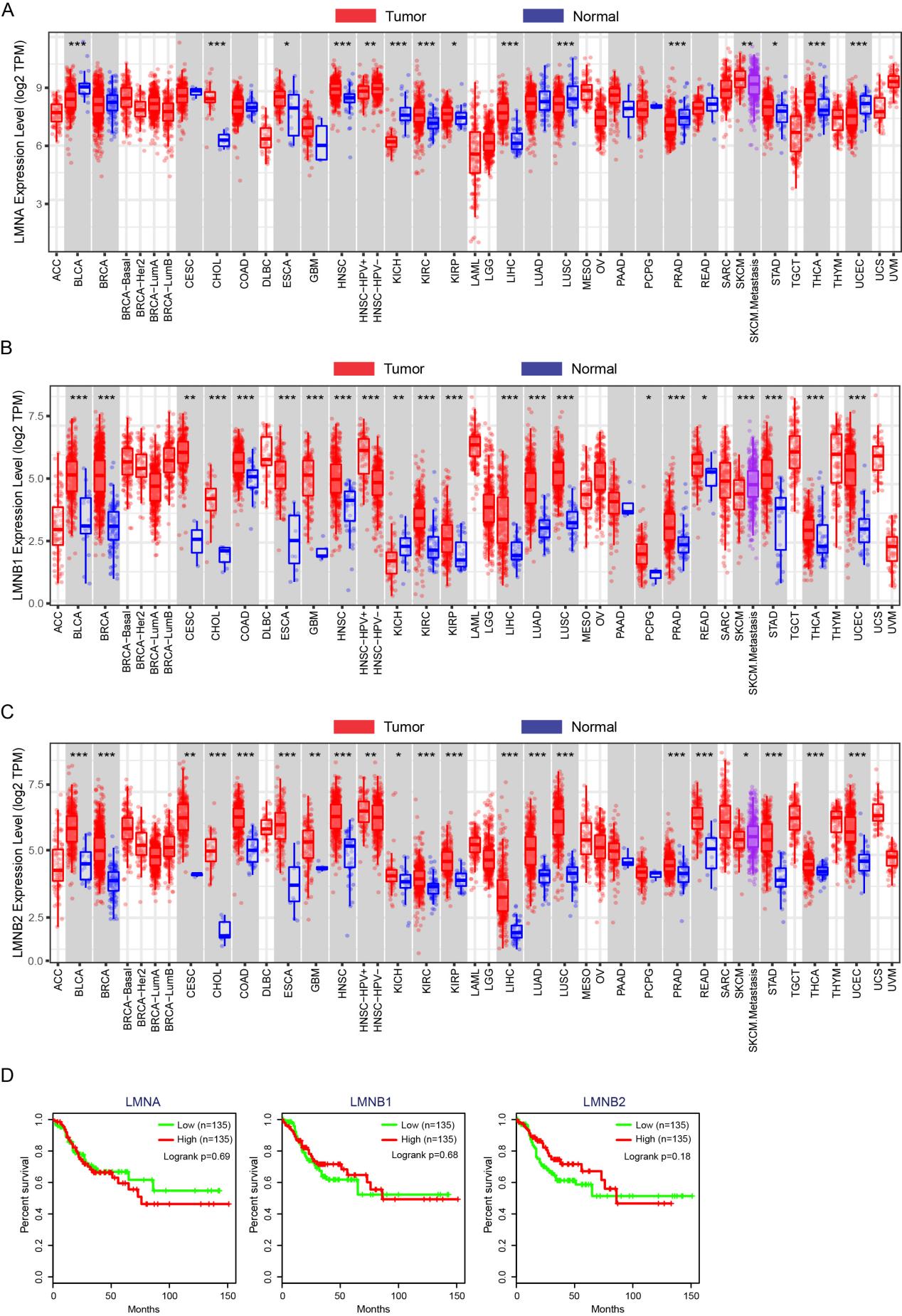


**Figure S1. Lamin transcripts are abnormally expressed in many human tumors.**

**(A-C)** Comparison of the expression levels of *LMNA* (A), *LMNB1* (B), and *LMNB2* (C) transcripts in various cancers and their adjacent normal tissues from the TCGA database. ACC: Adrenocortical carcinoma; BLCA: Bladder urothelial carcinoma; BRCA: Breast invasive carcinoma; CESC: Cervical and endocervical cancer; CHOL: Cholangiocarcinoma; COAD: Colon adenocarcinoma; DLBC: Diffuse large B-cell lymphoma; ESCA: Esophageal; GBM: Glioblastoma multiforme; HNSC: Head and neck squamous cell carcinoma; KICH: Kidney Chromophobe; KIRC: Kidney renal clear cell carcinoma; KIRP: Kidney renal papillary cell carcinoma; LGG: Lower grade glioma; LIHC: Liver hepatocellular carcinoma; LUAD: Lung adenocarcinoma; LUSC: Lung squamous cell carcinoma; OV: Ovarian serous cystadenocarcinoma; PAAD: Pancreatic adenocarcinoma; PCPG: Pheochromocytoma and paraganglioma; HNSC: Head and neck squamous cell carcinoma; PRAD: Prostate adenocarcinoma; READ: Rectum adenocacinoma; STAD: Stomachadenocarcinoma; SARC: Sarcoma; TGCT: Testicular germ cell tumors; THCA: Thyroid carcinoma; THYM: Thymoma; UCEC: Uterine corpus endometrial carcinoma. UCS: Uterine carsinosarcoma; UVM: Uveal melanoma. **(D)** Disease-free survival curves of cancer patients with either high or low expression level of *LMNA*, *LMNB1*, or *LMNB2*. Patient groups were classified by the median expression value of target transcripts within the study population.

**
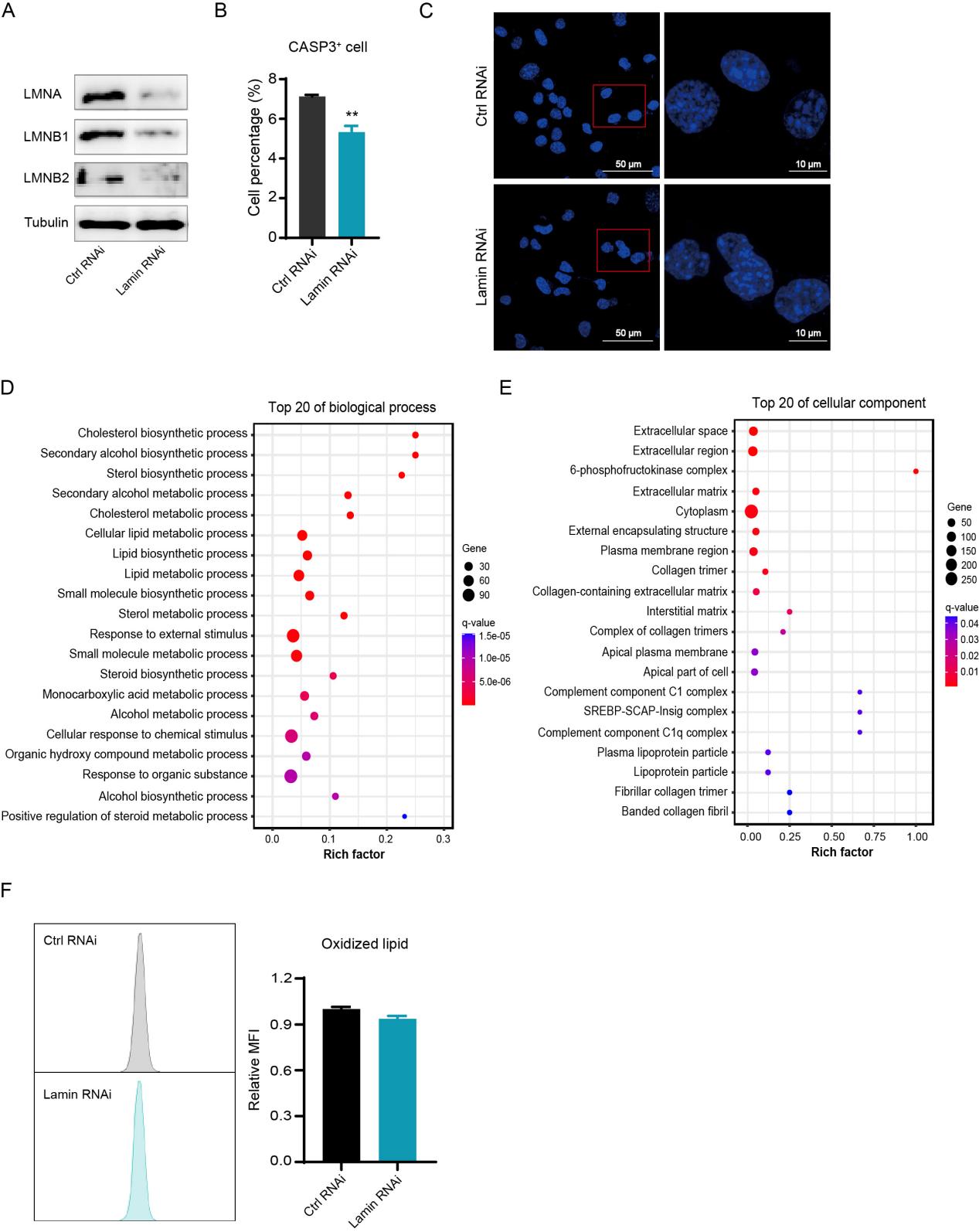
**

**Figure S2. Lamin knockdown alters nuclear shape and some cellular processes.**

(**A**) Western blotting analysis of LMNA, LMNB1, and LMNB2 expression levels in cancer cell without or with lamin knockdown. (**B**) FACS analysis of CASP3^+^ cell percentage in the control and lamin-deficient cancer cell. (**C**) Immunofluorescence analysis of nuclear morphology in the control and lamin-deficient cancer cell. Scale bars were shown in the images. (**D**) Top biological processes from RNA-seq data of cancer stem-like cell without or with lamin knockdown. (**E**) RNA-seq analysis of the altered cellular components in cancer cell after lamin knockdown. (**F**) FACS analysis of lipid oxidation in cancer cell without or with lamin knockdown. Left: Representative FACS histograms. Right: Relative fold change of the oxidized lipids. Quantification is shown as mean ± SEM (n = 3). ***P< 0.001 versus the control RNAi.

**
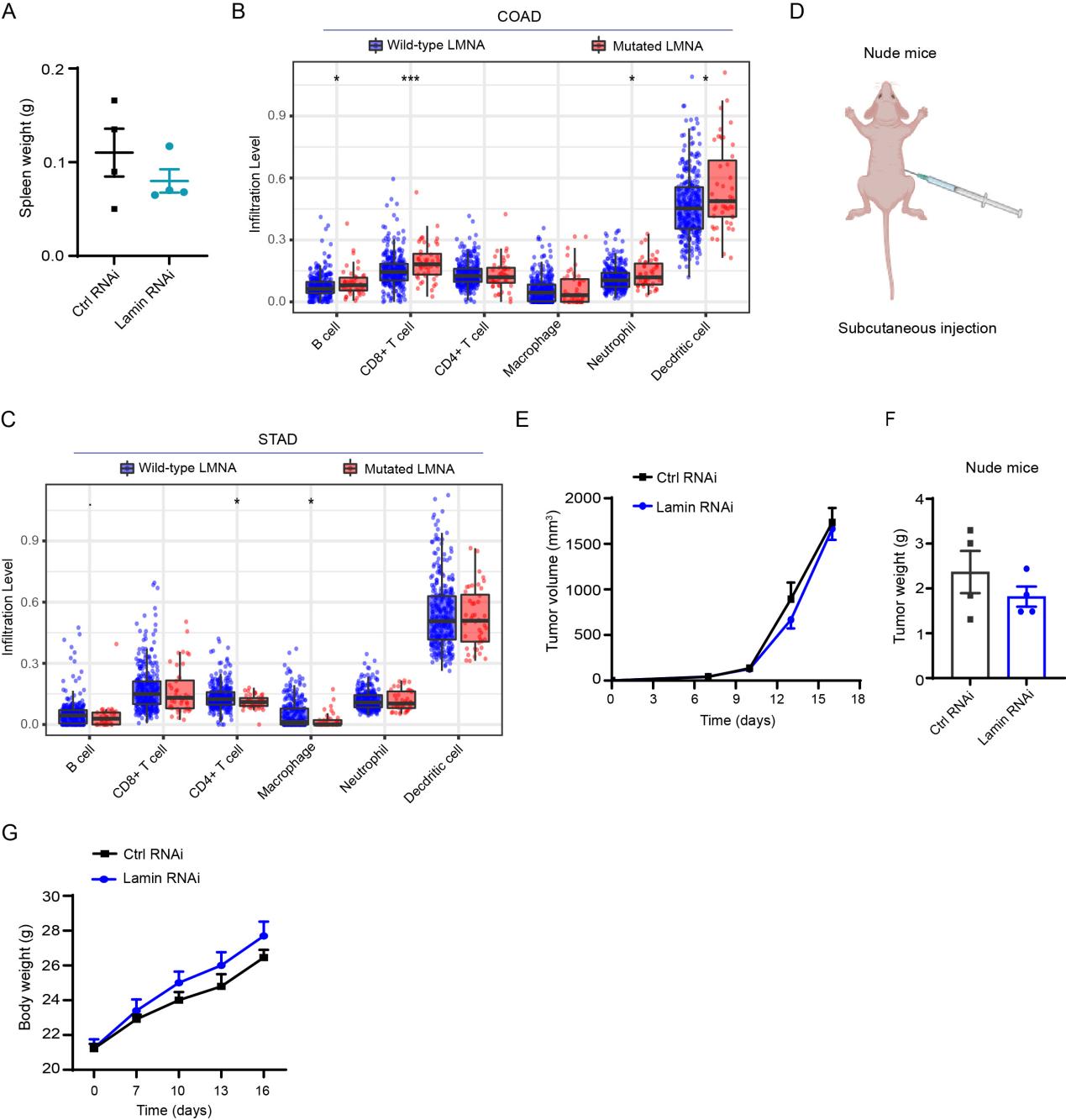
**

**Figure S3. Lamin knockdown does not impair tumor growth in nude mice.**

(**A**) The weights of spleens from BALB/c mice carrying the control or lamin-deficient tumors. (**B-C**) The correlation between LMNA mutation and the abundance of immune cells (B cell, CD4^+^ T cell, CD8^+^ T cell, Macrophage, Neutrophil, and Dendritic cell) in the COAD (B) or STAD (C). (**D**) Schematic diagram of the subcutaneous xenograft tumor model in nude mice. (**E**) The growth curves of control or lamin-deficient allografts in nude mice. (**F**) The weights of tumor allografts without or with lamin knockdown from nude mice. (**G**) Body weight changes of nude mice bearing the control or lamin-deficient allografts. Quantification is shown as mean ± SEM (n = 4).

**
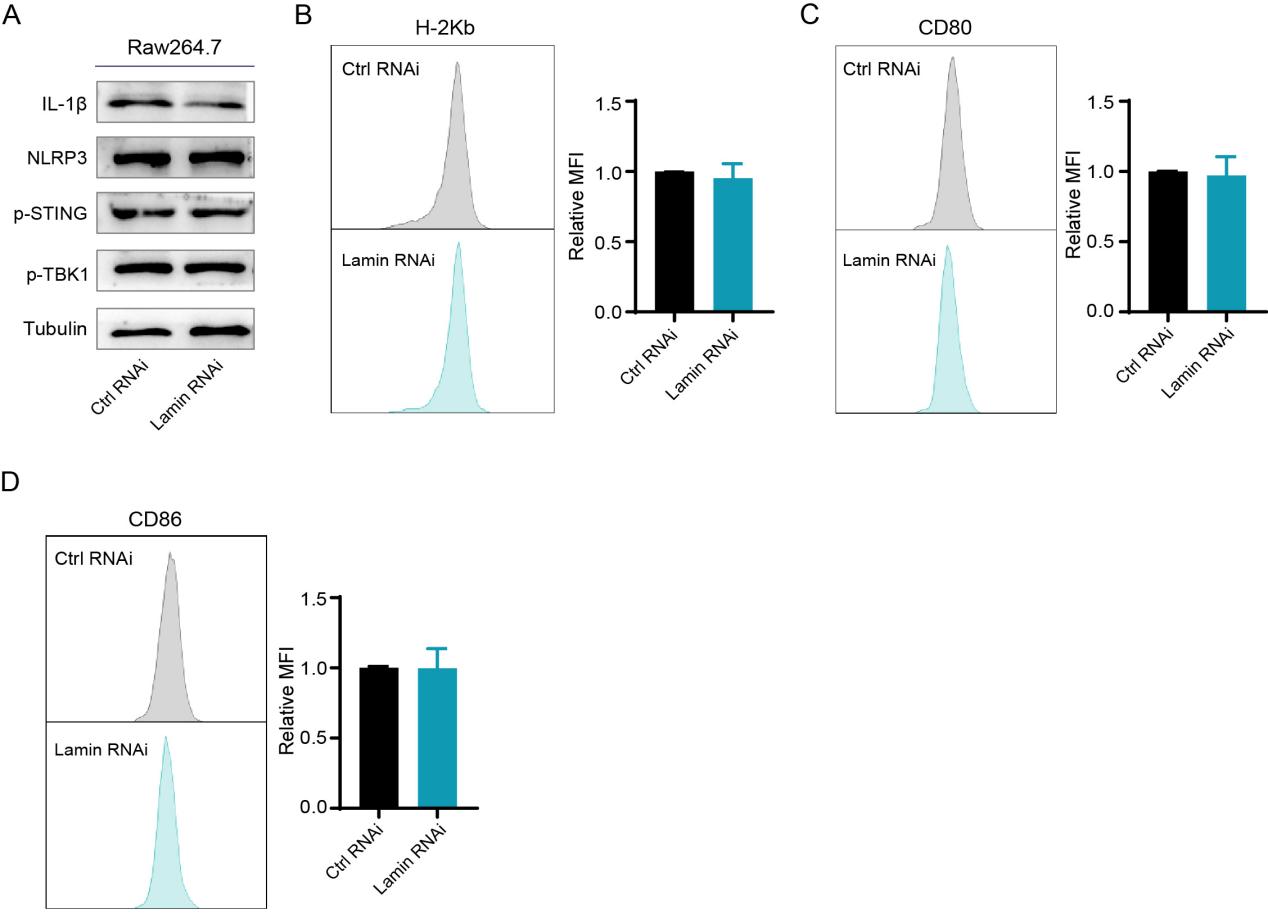
**

**Figure S4. Lamin knockdown in cancer cell does not activate macrophage.**

(**A**) Western blotting analysis of IL-1β, NLRP3, p-STING and p-TBK1 in the RAW264.7 cells treated with the conditional medium of control or lamin-deficient cancer cell. (**B**) FACS analysis of H-2Kb level on the RAW264.7 cells treated with the conditional medium of control or lamin-deficient cancer cell. Left: Representative FACS histograms; Right: Relative fold change of H-2Kb expression intensity. (**C**) FACS determination of CD80 level on the RAW264.7 cells treated with the conditional medium of cancer cell. Left: Representative FACS plots; Right: Quantification of relative fold changes. (**D**) FACS analysis of CD86 expression on the RAW264.7 cells treated with the conditional medium of cancer cell. Left: Representative FACS plots; Right: Quantification of relative fold change. Quantification is shown as mean ± SEM (n = 3).

**
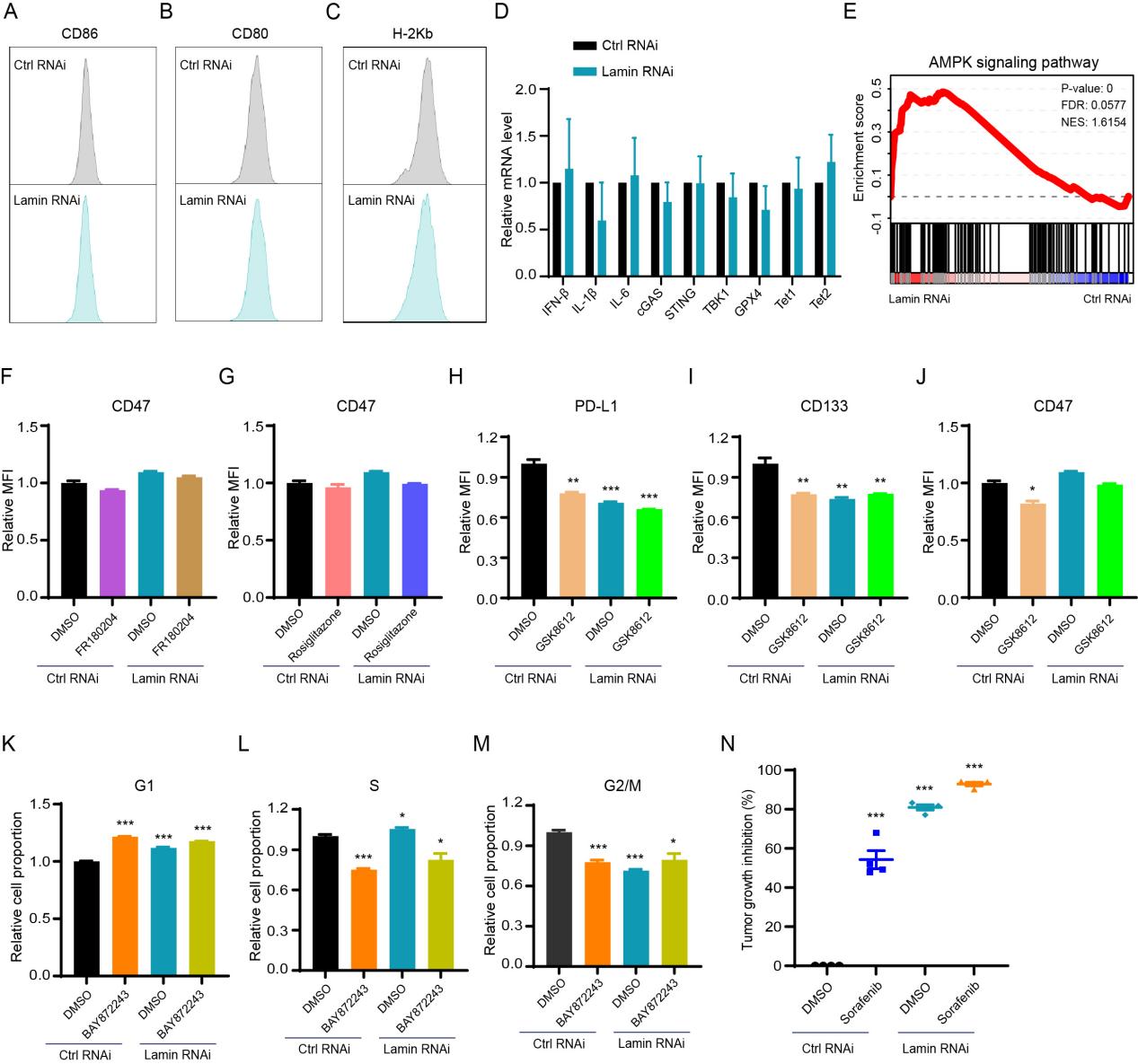
**

**Figure S5. Both PPAR-γ agonist and TBK1 inhibitor are unable to rescue the effects of lamin knockdown.**

**(A**) Representative FACS histograms of CD86 expression on control and lamin-deficient cancer cell. (**B**) Representative FACS plots of CD80 expression on cancer cell without or with lamin knockdown. (**C**) FACS determination of H-2Kb level on the control and lamin-deficient cell. (**D**) Q-PCR analysis of functional genes in cancer cell without or with lamin deficiency. (**E**) GSEA analysis of AMPK signaling pathway in the RNA-seq data of cancer stem-like cell without or with lamin knockdown. (**F**) FACS analysis of CD47 level on the control and lamin-deficient cell with DMSO or FR180204 treatment. **(G**) FACS determination of CD47 expression on the control and lamin-deficient cell without or with rosiglitazone treatment. (**H**) FACS analysis of PD-L1 expression on the control and lamin-deficient cell with DMSO or GSK8612 treatment. (**I**) FACS analysis of CD133 level on the control and lamin-deficient cell receiving DMSO or GSK8612 treatment. **(J**) FACS analysis of CD47 expression on the control and lamin-deficient cell without or with GSK8612 treatment. (**K-M**) Quantification of relative fold change of G1 (K), S (L), and G2/M (M) in the control and lamin-deficient cell treated with DMSO or BAY872243. (**N**) Quantification of reduction rate of lamin knockdown and/or sorafenib on the weights of xenograft tumors. Quantification is shown as mean ± SEM (n = 3). *P < 0.05, **P < 0.01, ***P< 0.001 versus the control RNAi or DMSO-treated control RNAi.

**
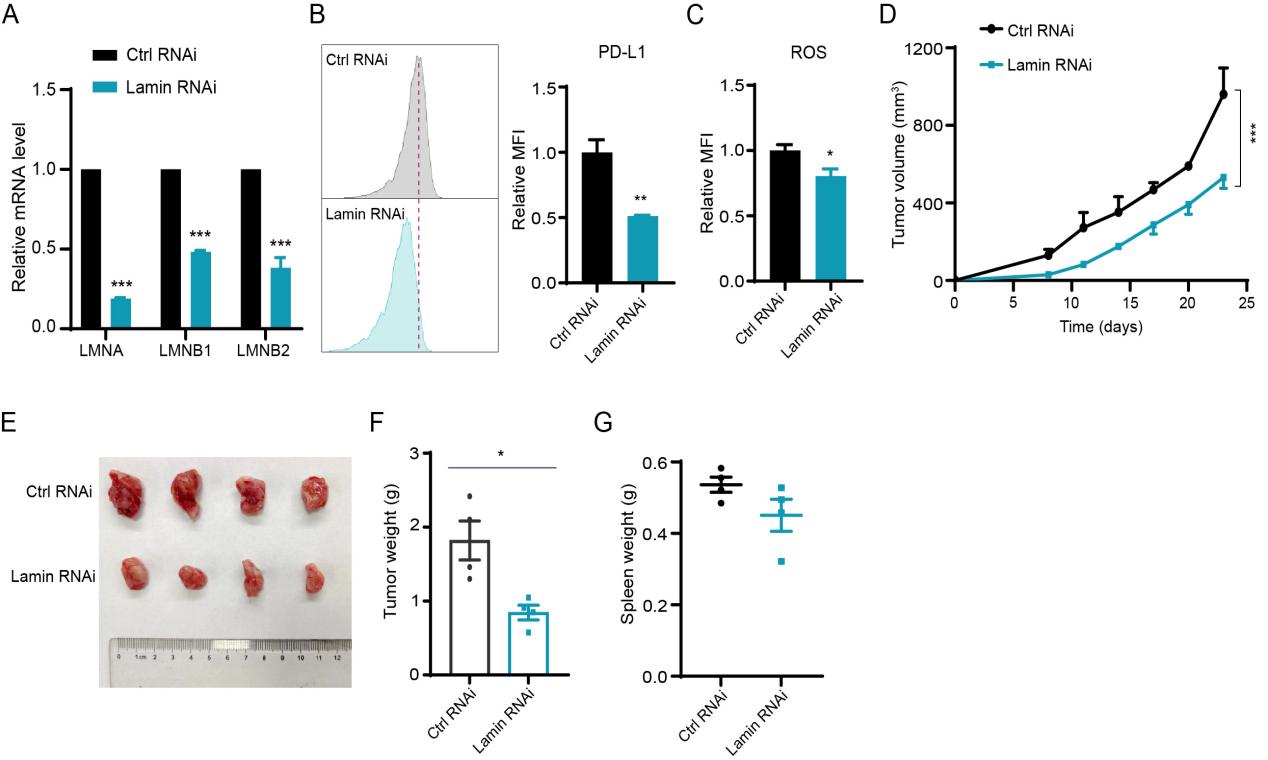
**

**Figure S6. Lamin knockdown in mammary cancer cell inhibits PD-L1 expression, ROS generation, and subcutaneous tumor growth.**

(**A**) Q-PCR analysis of *LMNA*, *LMNB1*, and *LMNB2* mRNA levels in mammary cancer cell without or with lamin knockdown. (**B**) FACS analysis of PD-L1 protein level in the control and lamin-deficient mammary cancer cell. Left: Representative FACS histograms; Right: Relative fold change of PD-L1 expression intensity. (**C**) FACS analysis of ROS level in the control and lamin-deficient cancer cell. (**D**) The growth curves of mammary xenograft tumors without or with lamin knockdown in BALB/c mice. (**E**) Representative images of mammary xenograft tumors without or with lamin knockdown. (**F**) Quantification of tumor weights as in (E). (**G**) The weights of spleens from BALB/c mice carrying the control or lamin-deficient mammary xenografts.

**
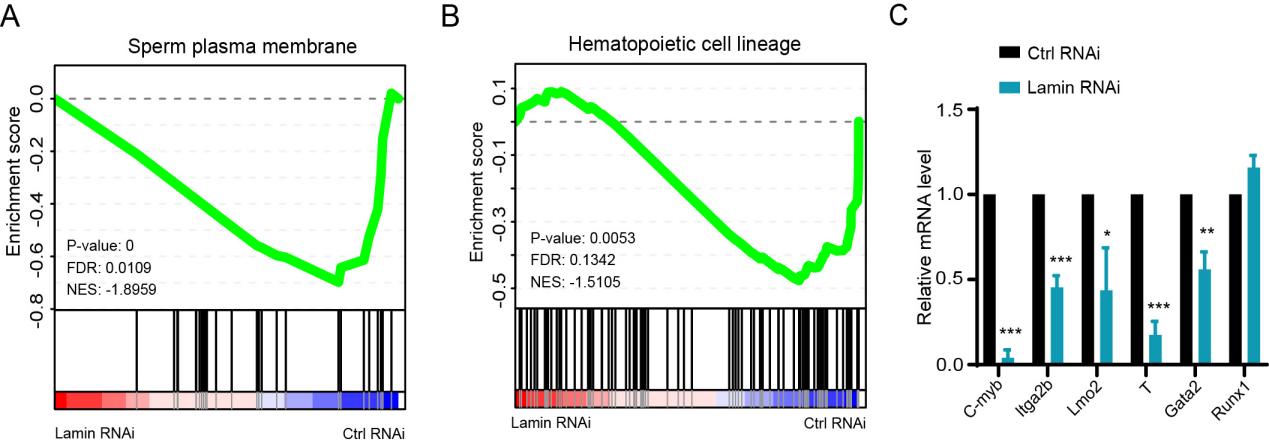
**

**Figure S7. All lamin knockdown likely inhibits hematopoietic differentiation.**

(**A**) GSEA analysis of sperm plasma membrane in the RNA-seq data of cancer stem-like cell. (**B**) GSEA analysis of hematopoietic lineage in the RNA-seq data of cancer stem-like cell without or with lamin knockdown. (**C**) Q-PCR analysis of several hematopoietic factors in cancer stem-like cells.

**Supplementary Table 1. The list of shRNA sequences.**

| **Name** | **Sequence (5' to 3')** |
| --- | --- |
| shLMNA-F | CCGGCCCACCGAAGTTCACCCTAAACTCGAGTTTAGGGTGAACTTCGGTGGGTTTTTG |
| shLMNA-R | AATTCAAAAACCCACCGAAGTTCACCCTAAACTCGAGTTTAGGGTGAACTTCGGTGGG |
| shLMNB1-F | CCGGGCGAATCTGATGGCCTTAATTCTCGAGAATTAAGGCCATCAGATTCGCTTTTTG |
| shLMNB1-R | AATTCAAAAAGCGAATCTGATGGCCTTAATTCTCGAGAATTAAGGCCATCAGATTCGC |
| shLMNB2-F | CCGGGCGTGACAAGTTCCGCAAGATCTCGAGATCTTGCGGAACTTGTCACGCTTTTTG |
| shLMNB2-R | AATTCAAAAACCCACCGAAGTTCACCCTAAACTCGAGTTTAGGGTGAACTTCGGTGGG |

**Supplementary Table 2**. **The sequences of RT-PCR primers.**

| **Primer** | **Sequence (5' to 3')** |
| --- | --- |
| LMNA-F | GGATGCTGAGAACAGGCTACA |
| LMNA-R | CTCTCGCTGCTTCCCGTTATC |
| LMNB1-F | CCGGCCTCAAGGCTCTCTA |
| LMNB1-R | TGCCGCCTCATACTCTCGAA |
| LMNB2-F | TGGCATCAAGACCCTGTACGA |
| LMNB2-R | TCAGCCTCACTCCGGTGAAA |
| IFNβ-F | CAGCTCCAAGAAAGGACGAAC |
| IFNβ-R | GGCAGTGTAACTCTTCTGCAT |
| IL1α-F | GCACCTTACACCTACCAGAGT |
| IL1α-R | AAACTTCTGCCTGACGAGCTT |
| IL1β-F | GCTGCTTCCAAACCTTTGACC |
| IL1β-R | GGTGCTCATGTCCTCATCCTGG |
| IL6-F | TAGTCCTTCCTACCCCAATTTCC |
| IL6-R | TTGGTCCTTAGCCACTCCTTC |
| cGAS-F | GAGGCGCGGAAAGTCGTAA |
| cGAS-R | TTGTCCGGTTCCTTCCTGGA |
| STING-F | GGTCACCGCTCCAAATATGTAG |
| STING-R | CAGTAGTCCAAGTTCGTGCGA |
| TBK1-F | ACTGGTGATCTCTATGCTGTCA |
| TBK1-R | TTCTGGAAGTCCATACGCATTG |
| GPX4-F | GATGGAGCCCATTCCTGAACC |
| GPX4-R | CCCTGTACTTATCCAGGCAGA |
| Tet1-F | ACACAGTGGTGCTAATGCAG |
| Tet1-R | AGCATGAACGGGAGAATCGG |
| Tet2-F | AGAGAAGACAATCGAGAAGTCGG |
| Tet2-R | CCTTCCGTACTCCCAAACTCAT |
| CCL2-F | TAAAAACCTGGATCGGAACCAAA |
| CCL2-R | GCATTAGCTTCAGATTTACGGGT |
| CCL3-F | AGTTCTCTGCATCACTTGCTG |
| CCL3-R | CGGCTTCGCTTGGTTAGGAA |
| CCL4-F | CTGTGCTGATCCCAGTGAATC |
| CCL4-R | TCAGTTCAGTTCCAGGTCATACA |
| CCL5-F | GCTGCTTTGCCTACCTCTCC |
| CCL5-R | TCGAGTGACAAACACGACTGC |
| CCL6-F | GCTGGCCTCATACAAGAAATGG |
| CCL6-R | GCTTAGGCACCTCTGAACTCTC |
| CCL7-F | GCTGCTTTCAGCATCCAAGTG |
| CCL7-R | CCAGGGACACCGACTACTG |
| CCL9-F | CCCTCTCCTTCCTCATTCTTACA |
| CCL9-R | AGTCTTGAAAGCCCATGTGAAA |
| CCL11-F | GAATCACCAACAACAGATGCAC |
| CCL11-R | ATCCTGGACCCACTTCTTCTT |
| CCL22-F | ATTACGTCCGTTACCGTCTGC |
| CCL22-R | TCCCTGAAGGTTAGCAACACC |
| CCL25-F | TTACCAGCACAGGATCAAATGG |
| CCL25-R | CGGAAGTAGAATCTCACAGCAC |
| CCL28-F | GTGTGTGGCTTTTCAAACCTCA |
| CCL28-R | TGCATGAACTCACTCTTTCCAG |
| CXCL1-F | CTGGGATTCACCTCAAGAACATC |
| CXCL1-R | CAGGGTCAAGGCAAGCCTC |
| CXCL5-F | TCCAGCTCGCCATTCATGC |
| CXCL5-R | TTGCGGCTATGACTGAGGAAG |
| CXCL9-F | GGAGTTCGAGGAACCCTAGTG |
| CXCL9-R | GGGATTTGTAGTGGATCGTGC |
| CXCL10-F | ATGACGGGCCAGTGAGAATG |
| CXCL10-R | TCAACACGTGGGCAGGATAG |
| CXCL11-F | GACGCTGTCTTTGCATAGGC |
| CXCL11-R | GGATTTAGGCATCGTTGTCCTTT |
| CXCL12-F | ATTCTCAACACTCCAAACTGTGC |
| CXCL12-R | ACTTTAGCTTCGGGTCAATGC |
| CXCL13-F | CTCCAGGCCACGGTATTCTG |
| CXCL13-R | CCAGGGGGCGTAACTTGAAT |
| XCL1-F | TTTGTCACCAAACGAGGACTAAA |
| XCL1-R | CCAGTCAGGGTTATCGCTGTG |
| IFNAR1-F | AGCCACGGAGAGTCAATGG |
| IFNAR1-R | GCTCTGACACGAAACTGTGTTTT |
| IFNGR2-F | CCGAACTGTACGGACATCACA |
| IFNGR2-R | TCATAGTGTTGAAATGGCTCCAG |
| IFNGR1-F | TGACTATGCACGGTCAAAAGAG |
| IFNGR1-R | ATTCACAACGACTTCAGGGTG |
| GAPDH-F | AGGTCGGTGTGAACGGATTTG |
| GAPDH-R | TGTAGACCATGTAGTTGAGGTCA |
